# Synergistic Interactions Among Iron and Cobalt Atoms Within Bimetallic Molybdate@Carbon Paper Composite Create Bifunctional Nanoflower Electrocatalyst, Enhancing Efficiency for Overall Water Splitting in Alkaline Environment

**DOI:** 10.3390/molecules30040844

**Published:** 2025-02-12

**Authors:** Ting Cheng, Fei Wu, Chen Chen, Xiao Zhang, Mengyi Zhang, Liwei Cui, Youzhi Dai, Baoxuan Hou, Yuan Tian, Jiarui Zhu

**Affiliations:** 1 School of Environmental Ecology, The City Vocational College of Jiangsu, Nanjing 210017, China; wnchengting@sina.com (T.C.); zhangxiao7376@sina.com (X.Z.); 2 Jiangsu Engineering and Technology Centre for Ecological and Environmental Protection in Urban and Rural Water Environment Management and Low Carbon Development, Nanjing 210017, China; 3 School of Environmental and Chemical Engineering, Jiangsu University of Science and Technology, Zhenjiang 212100, China; wufei1224wf@hotmail.com (F.W.); zhangmengyi031029@163.com (M.Z.); cui36984628@163.com (L.C.); hbx.1999@outlook.com (B.H.); ttyy1974.ok@163.com (Y.T.); zjr000323@163.com (J.Z.); 4 Nanjing University and Yancheng Academy of Environmental Protection Technology and Engineering, Yancheng 224000, China; 5 College of Environment and Resource, Xiangtan University, Xiangtan 411105, China; daiyouzhi202@163.com

**Keywords:** HER, OER, overall water splitting, molybdate, cobalt, iron

## Abstract

Electrocatalytic water splitting is a promising approach for obtaining clean hydrogen energy. In this work, novel molybdate@carbon paper composite electrocatalysts (CoxFe10-xMoO@CP), displaying outstanding electrocatalytic capabilities, were deriving from anchoring cobalt/iron molybdate materials onto the surface of carbon paper fibers. By adjusting the cobalt-to-iron ratio, the composite (Co5Fe5MoO@CP), with the optimal molar proportion (Co/Fe = 5/5), exhibited a distinctive nanoflower morphology (50–100 nm), which provided a significant number of active sites for electrocatalytic reactions, and showed the strongest electrocatalytic potency for both the hydrogen evolution reaction (HER) and oxygen evolution reaction (OER). Specifically, the overpotentials for HER and OER were 123.6 and 245 mV at 10 mA·cm^−2^, with a Tafel slope of 78.3 and 92.2 mV·dec^−1^, respectively. The hydrogen and oxygen evolution reactions remained favorable and stable over 35 days and 2 weeks of cyclic voltammetry cycles. In a two-electrode system, efficient overall water splitting was achieved at a cell voltage of 1.60 V. Under high alkaline concentration and temperature conditions, the Co5Fe5MoO@CP composite still maintained excellent HER and OER catalytic activity and stability, indicating its satisfactory potential for industrial applications. Density functional theory (DFT) analysis revealed that the promoted hydrogen evolution capability derived from the synergistic catalytic effect of iron and cobalt atoms within the molecule, while cobalt atoms functioned as the catalytic core for the oxygen evolution process. This work provides a novel strategy towards high-efficiency electrocatalysts to significantly accelerate the overall water splitting.

## 1. Introduction

Fossil fuels have played a crucial role in advancing human civilization. However, their non-renewable nature and the pollution they generate—impacting the atmosphere, water, and soil—have considerably hindered global sustainable development [1,2]. To address this challenge, research on green energy sources—including wind [2,3], solar [4,5,6], water [7], nuclear [8,9], and hydrogen energy [10,11]—has become a central focus in the fields of energy and materials science. Among these green energy sources, hydrogen energy stands out as the most promising due to its combustion product being solely water, thus rendering it completely pollution-free. Presently, the overall water splitting via electrocatalysis is widely acknowledged as a straightforward, efficient, and dependable method for hydrogen production [12,13].

Electrocatalytic overall water splitting is a conventional method for producing hydrogen and oxygen, involving a hydrogen evolution reaction (HER) at the cathode and an oxygen evolution reaction (OER) at the anode [14,15]. Given that the standard potential difference between HER and OER is 1.23 V, theoretically, electrocatalytic water splitting can occur when the cell voltage exceeds 1.23 V. However, due to overpotentials [16,17], the actual voltage required is higher, which significantly reduces the economic feasibility of the process.

To enhance the viability of electrocatalytic hydrogen production, extensive research has focused on developing efficient electrocatalysts to lower the overpotentials of HER and OER [18,19]. Precious metal electrocatalysts, such as Pt/C, RuO_2_, and IrO_2_, are currently considered highly efficient. Nevertheless, the use of noble metals like Pt [20,21], Ru [22,23], and Ir [24,25] greatly increase the cost of hydrogen production. Although promising, the industrial application of these materials at scale is seriously constrained by their expense and scarcity. Additionally, a single noble metal catalyst faces challenges in simultaneously boosting HER and OER efficiencies, and their stabilities under strong alkaline conditions are typically suboptimal. Consequently, creating cost-effective, durable, stable, and highly active bifunctional catalysts is vital to overcoming these challenges and advancing overall water splitting [26] and that has become a prominent research focus in the field of green energy.

Up to this point, research into non-noble catalysts has mainly revolved around transition metals, with cobalt and iron being the primary focus. Numerous studies have revealed that cobalt- and iron-based materials are preferred as low-cost HER and OER bifunctional catalysts. For example, Sung et al. synthesized CeO_2_/Co(OH)_2_, achieving the overpotentials for the HER and OER of 317 mV and 410 mV, respectively, at a current density of 10 mA·cm^−2^, with Tafel slopes of 140 and 66 mV·dec^−1^ [27]. Further research has shown that composite materials incorporating these two transition elements and molybdenum often exhibit superior catalytic performance, and these element-based composite catalysts demonstrate significant promise in boosting HER and OER efficiency. For instance, Alkhaldi et al. synthesized a ZnCo_1.94_Mo_0.06_O_4_ catalyst, which demonstrated an HER overpotential of 195 mV with a Tafel slope of 81.4 mV·dec^−1^ at a current density of 10 mA·cm^−2^ [28]. Additionally, Zhang et al. prepared the SrCoFe_0.4_Mo_0.1_O_3−δ_ catalyst, achieving HER and OER overpotentials of 200 mV and 310 mV, respectively, at a current density of 10 mA·cm^−2^ [29]. Nonetheless, the increase in water splitting efficiency through the straightforward integration of two or more separate components, lacking intrinsic electronic interaction at the interface, is generally constrained. It is broadly acknowledged that excellent electrocatalytic materials require not only outstanding theoretical catalytic performance but also superior electron transfer capabilities [30,31].

It is considered that effective electronic transmission is critical for a top-tier electrocatalyst. The efficiency of electron transfer is largely determined by the choice of a highly conductive substrate material for the catalysts. To enhance electron transfer capabilities, a feasible way is to load active catalytic materials onto conductive substrates, such as nickel foam, carbon cloth, and carbon paper. For instance, Wang et al. successfully synthesized Co-MoS_2_ on nitrogen-doped carbon fiber/carbon paper, achieving efficient electrocatalytic activity with an HER overpotential of 174 mV and a Tafel slope of 52 mV·dec^−1^ at 10 mA·cm^−2^ [32]. Similarly, Li et al. developed Co-N-C directly grown on carbon fiber paper, which exhibited excellent electrocatalytic performance with an HER overpotential of 181 mV and OER overpotential of 290 mV at 10 mA·cm^−2^ [33]. As a result, it is essential to develop catalysts that combine remarkable intrinsic activity with well-defined external physical and chemical properties. So far, the available experimental findings for the formation of low-cost, highly efficient, durable, bifunctional catalysts are inadequate, and the catalytic mechanisms associated with these catalysts are not well understood, necessitating further clarification. Also, there is a pressing need for the development of highly efficient and stable bifunctional electrocatalysts to boost both the HER and OER process, accelerate the electrocatalytic overall water splitting, and achieve the industrial applications.

Building upon the aforementioned analysis and previous research, this study innovatively synthesized iron/cobalt molybdenum oxide (CoxFe10−xMoO) via a hydrothermal method. Subsequently, a self-supporting electrocatalyst of CoxFe10−xMoO@CP was successfully produced by anchoring CoxFexMoO onto carbon paper. By adjusting the ratio of cobalt to iron, the microstructure of the electrocatalyst materials was altered accordingly, resulting in the outstanding bifunctional electrocatalytic capability of composite electrocatalyst (Co5Fe5MoO@CP), with distinctive nanoflower morphology and a significant number of active sites for electrocatalytic reactions. These materials exhibited optimal electrocatalytic properties and superior stability for HER and OER and greatly enhanced the overall water splitting efficiencies. Density functional theory (DFT) calculations revealed that the vigorous electrocatalytic capability of HER was ascribed to the synergistic catalytic interactions between iron and cobalt atoms in Co5Fe5MoO@CP composite, and cobalt atoms acted as the catalytic center for the oxygen evolution process. This research offers a fresh perspective for achieving high-performance electrocatalysts that can markedly speed up the overall water splitting process, presenting a promising prospect for industrial implementation.

## 2. Experiments and Methods

**Figure 1:**
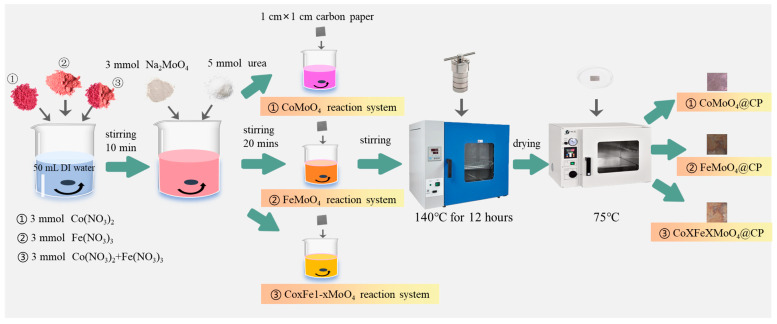
The synthesis process of CoMoO@CP, FeMoO@CP, and CoxFe10-xMoO@CP materials.

The chemical reagents utilized in this study were of analytical grade and procured from Sigma Chemical Reagent Co., Ltd. (Shanghai, China). Carbon paper (model: TGP-H-060; brand: Toray) was obtained from Suzhou Sinero Technology Co., Ltd. (Suzhou, China). The material synthesis process was illustrated in Figure 1. The synthesis of CoMoO@CP material via hydrothermal method was conducted as follows: Initially, 3 mmol of cobalt nitrate (Co(NO_3_)_2_, Sigma Chemical Reagent Co., Ltd., Shanghai, China) was dissolved in 50 mL of deionized water, and the solution was stirred for 30 min. Subsequently, 3 mmol of sodium molybdate (Na_2_MoO_4_, Sigma Chemical Reagent Co., Ltd., Shanghai, China) and 5 mmol of urea (Sigma Chemical Reagent Co., Ltd., Shanghai, China) were sequentially introduced into the solution, which was then stirred for an additional 30 min until complete dissolution of the reagents. A carbon paper substrate (1 cm × 1 cm) was then immersed into the solution, which continued to be stirred for another 40 min. The resulting mixture was transferred to a 100 mL stainless steel autoclave lined with Teflon and subjected to hydrothermal treatment at 140 °C for 12 h.

Upon completion of the reaction, the reactor was allowed to cool slowly to room temperature before being opened to retrieve the carbon paper composite material. The resulting CoMoO@CP composite was then dried at a constant temperature of 75 °C until a constant weight was achieved. For the synthesis of FeMoO@CP materials, the procedure was similar to that described above, with cobalt nitrate (Co(NO_3_)_2_) being substituted with 3 mmol of iron nitrate (Fe(NO_3_)_3_, Sigma Chemical Reagent Co., Ltd., Shanghai, China). Composite materials containing both cobalt and iron in various ratios were synthesized in an analogous manner. The ratios of Co to Fe for these materials were 2:8, 3:7, 4:6, 5:5, 6:4, 7:3, and 8:2, with the total amount of cobalt and iron being 3 mmol. These materials were designated as CoxFe10-xMoO@CP (where x:10-x represented the molar ratio of cobalt to iron in the synthesis system), for example, Co2Fe8MoO@CP indicated a molar ratio of Co to Fe of 2:8 in the composite catalyst. Prior to XRD and TEM analyses, the active components of the composite materials were isolated by ultrasonic dispersion and were labeled as CoxFe10-xMoO. Prior to conducting the materials’ characterization, the active substances of the composite material were isolated via ultrasonic oscillation and designated as CoxFe10-xMoO.

All electrochemical experiments were conducted at 25 °C, with temperature control maintained by laboratory air conditioning, using a CHI 660E electrochemical workstation (Shanghai Chenhua, Shanghai, China). The electrochemical evaluations for hydrogen evolution reaction (HER) and oxygen evolution reaction (OER) were performed using a conventional three-electrode configuration. The electrolyte utilized was a 1 M KOH (Sigma Chemical Reagent Co., Ltd., Shanghai, China) solution. The synthesized carbon paper composite material was employed directly as the working electrode. The counter electrode consisted of a graphite rod, and the reference electrode was a mercury/mercury oxide electrode. During data analysis, the potential measurements were converted to the reversible hydrogen electrode (RHE) scale. The relationship between the test potential and the standard reversible hydrogen electrode potential (RHE) was represented by Equation (1).

(1) $$E_{RHE}=E_{Hg/HgO}+0.098+0.0591pH$$

Initially, the electrolyte was purged with high-purity nitrogen for 40 min to remove dissolved oxygen. The electrochemical active surface area was assessed by scanning cyclic voltammetry (CV) curves in the non-Faraday region, with potential ranges of −0.06 to −0.02 V or −0.04 to 0 V (vs. Hg/HgO) and scan rates ranging from 10 to 100 mV/s. Subsequently, the HER and OER curves were analyzed using IR-corrected linear sweep voltammetry (LSV). For HER, the LSV was conducted within a potential range of −1.92 to −0.92 V (vs. Hg/HgO) at a scan rate of 2 mV/s. For OER, the LSV was performed within a potential range of 0 to 2 V (vs. Hg/HgO) at a scan rate of 5 mV/s. The Tafel slopes were determined by fitting data from the steady-state portions of the HER and OER curves. The electrochemical impedance spectroscopy (EIS) was conducted for both HER and OER over a frequency range of 0.1 to 1000 Hz with an amplitude of 5 mV. The testing potential was selected from the LSV curve corresponding to a current density of 10 mA·cm^−2^.

The resulting impedance spectral data were analyzed and fitted to appropriate equivalent circuits using specialized software (ZView 3.1). To assess the electrocatalytic stability, current density–time (i-t) curves for both HER and OER were recorded over a duration of 35 days, with the testing potential selected from the LSV curve corresponding to a current density of 10 mA·cm^−2^. Additionally, the IR-corrected LSV curves for both HER and OER were re-measured after 2 weeks of cyclic voltammetry to evaluate the catalytic stability. For the overall water splitting experiment, conditions were consistent with those used for individual HER and OER tests. The synthesized carbon paper composite material served as both the working and counter electrodes in a dual-electrode system. Linear sweep voltammetry (LSV) was conducted at a scan rate of 2 mV/s, covering a cell voltage range of 0 to 2.5 V. In the investigation of industrial application potential, the KOH electrolyte concentration for LSV testing (HER and OER) included 1, 2, 4, and 6 M. The reaction temperatures included 25, 60, 70, and 80 °C. The current density–time (i-t) curves for both HER and OER were recorded over a span of 35 days under 6 M KOH and 70 °C. Other detailed descriptions of the material characterization and density functional theory calculations were provided in the Supplementary Material.

## 3. Results and Discussion

### 3.1. XRD

**Figure 2:**
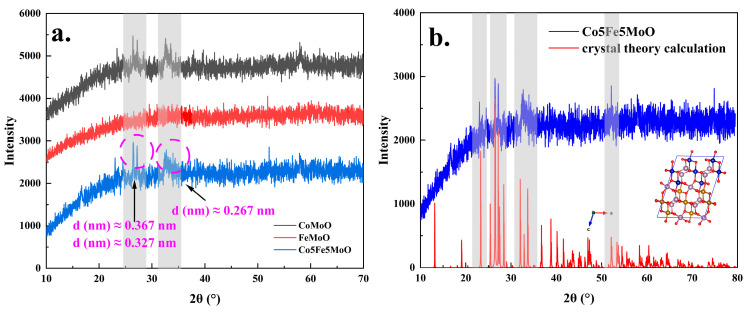
The XRD analysis results of CoMoO, FeMoO, and Co5Fe5MoO powder materials (**a**); the comparison of XRD patterns of actual Co5Fe5Mo and theoretical crystal structure (**b**).

Figure 2a presented the XRD analysis results for CoMoO, FeMoO, and Co5Fe5MoO materials. The XRD patterns for all three materials revealed the absence of pronounced crystal plane diffraction peaks, suggesting that the synthesized cobalt/iron molybdate materials predominantly exhibit a weak crystal structure. Similar findings were observed for materials with other cobalt/iron ratios (see Figure S9), indicating a consistent formation of weak crystalline structures. Although no strong peaks are present, several weaker diffraction peaks were discernible in the XRD patterns of CoMoO and Co5Fe5MoO, specifically around 2θ values of 26.4°, 27.2°, and 32.7°. Calculations indicated that the interplanar spacings corresponding to these XRD peaks were approximately 0.367 nm, 0.327 nm, and 0.267 nm, respectively. The analysis of the XRD results for materials with varying cobalt/iron ratios revealed that the appearance of weak peaks was associated with the incorporation of cobalt, with peak intensity increasing proportionally to the cobalt content (Figure S9). This observation suggested that changes in the cobalt/iron ratio influenced the crystal structure of the molybdate materials, which might, in turn, impact their electrocatalytic performance. Figure 2b compared the XRD results of the actual Co5Fe5MoO with theoretical crystal structure calculations. Although the weaker peaks were not observable due to the formation of a predominantly weak crystal structure, the positions and relative intensities of several prominent peaks aligned closely with the theoretical predictions. Consequently, the subsequent DFT calculations would be based on the theoretical crystal structures depicted in Figure 2b (insert) and Figure S1.

### 3.2. SEM and TEM

**Figure 3:**
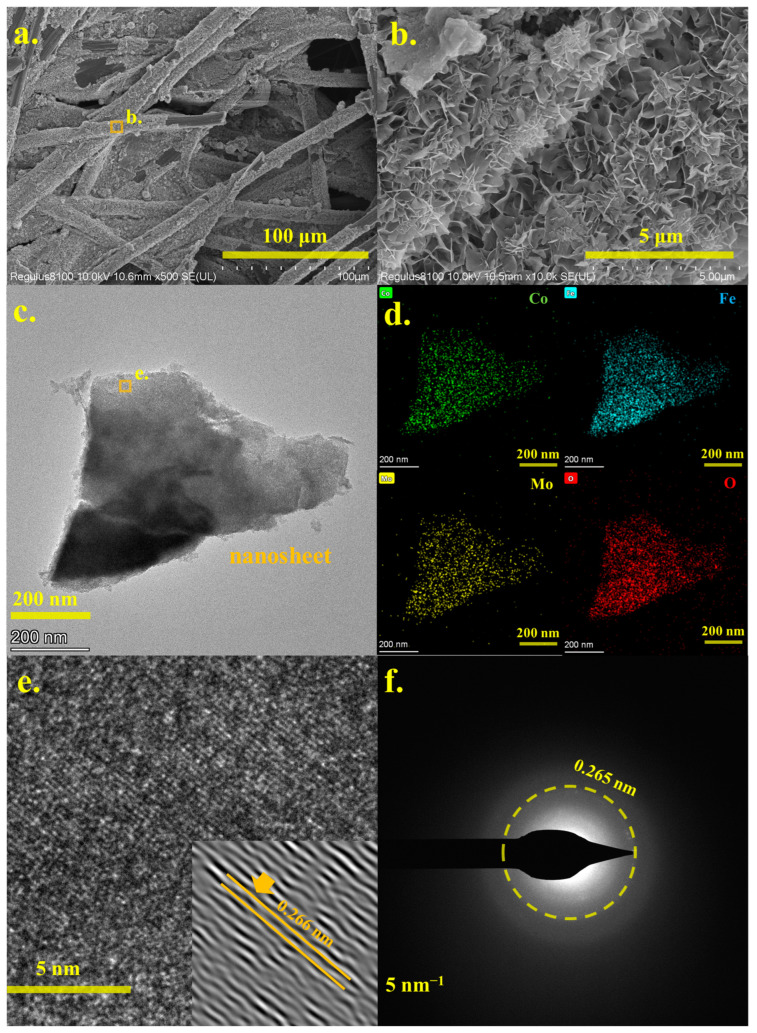
The SEM analysis results of Co5Fe5MoO@CP composite (**a**,**b**); the TEM and related EDX mapping analysis results of Co5Fe5MoO@CP (**c**,**d**); HRTEM and diffraction ring of Co5Fe5MoO@CP (**e**,**f**).

Micro-morphology was a crucial property of electrocatalytic materials, and Figure 3 presented the SEM and TEM analysis results for the Co5Fe5MoO@CP composite. As shown in Figure 3a, following the hydrothermal synthesis, the surface of the carbon paper fibers was covered with a substantial layer of reaction products. This coating morphology probably enhanced the electron transfer between the carbon paper fibers and the active catalytic surfaces during the electrocatalytic processes. Upon higher magnification (see Figure 3b and Figure S10), the reaction products clearly exhibited a nanoflower morphology. The nanoflower sheets were approximately 50–100 nm in thickness and 1–2 μm in length. This distinctive morphology provided a significant number of active sites for electrocatalytic reactions. The subsequent SEM wide-range EDX mapping analysis (see Figures S11 and S12) revealed that the primary elemental composition of the areas shown in Figure 3b and Figures S11 and S12 included cobalt, iron, molybdenum, oxygen, and carbon, which aligned with the elemental composition of both carbon paper and the Co5Fe5MoO material. More precise analytical results were obtained through TEM analysis, as illustrated in Figure 3c–f. Figure 3c clearly displayed the morphology of a single nanoflower sheet, with side lengths ranging from approximately 200 nm to 1 μm. TEM–EDX analysis further confirmed that cobalt, iron, molybdenum, and oxygen were uniformly distributed within the nanoflower particles, corroborating the presence of these four elements. High-resolution TEM (HRTEM) images and diffraction ring patterns of the Co5Fe5MoO@CP material were shown in Figure 3e,f. The HRTEM results revealed distinct diffraction fringes corresponding to a crystal plane spacing of 0.266 nm, which aligned closely with the peak observed at approximately 32.7° in the XRD results (Figure 2a). Similar findings could also be obtained from Figure 3f, and the clear diffraction ring belonging to 0.265 nm could be observed. These results further supported that the nanoflower sheets possessed the theoretical crystal structure depicted in Figure S1.

In addition, Figures S13–S18 presented the SEM analysis results for FeMoO@CP, Co3Fe7MoO@CP, and CoMoO@CP materials. As observed in Figures S13 and S14, when the reaction system included only iron, molybdenum, and oxygen, the resulting products exhibited a blocky micro-morphology rather than the desired nanoflower sheets. Consequently, FeMoO@CP had a relatively low catalytic surface area, which was disadvantageous for electrocatalytic processes. As the cobalt content in the composite increased, the Co3Fe7MoO@CP composite began to display some nanosheet morphology (see Figure S15), though it had not yet formed the abundant nanoflower sheets seen in the Co3Fe7MoO@CP composite. When the reaction system consisted solely of cobalt, molybdenum, and oxygen, nanosheets were still present in the products (Figures S17 and S18); however, they tended to agglomerate on the carbon paper surface (Figure S17) rather than coating the carbon paper fibers as observed with Co3Fe7MoO@CP (Figure 3b). Since carbon paper fibers served as the medium for electron transfer during catalysis, this morphology was less conducive to effective electron transfer to the catalytic active sites. Overall, it was evident that the composition of cobalt and iron in the hydrothermal reactions directly influenced the micro-morphology of the reaction products. For electrocatalytic applications, the Co3Fe7MoO@CP composite exhibited more favorable micro-morphological characteristics compared to CoMoO@CP and FeMoO@CP materials.

### 3.3. XPS

**Figure 4:**
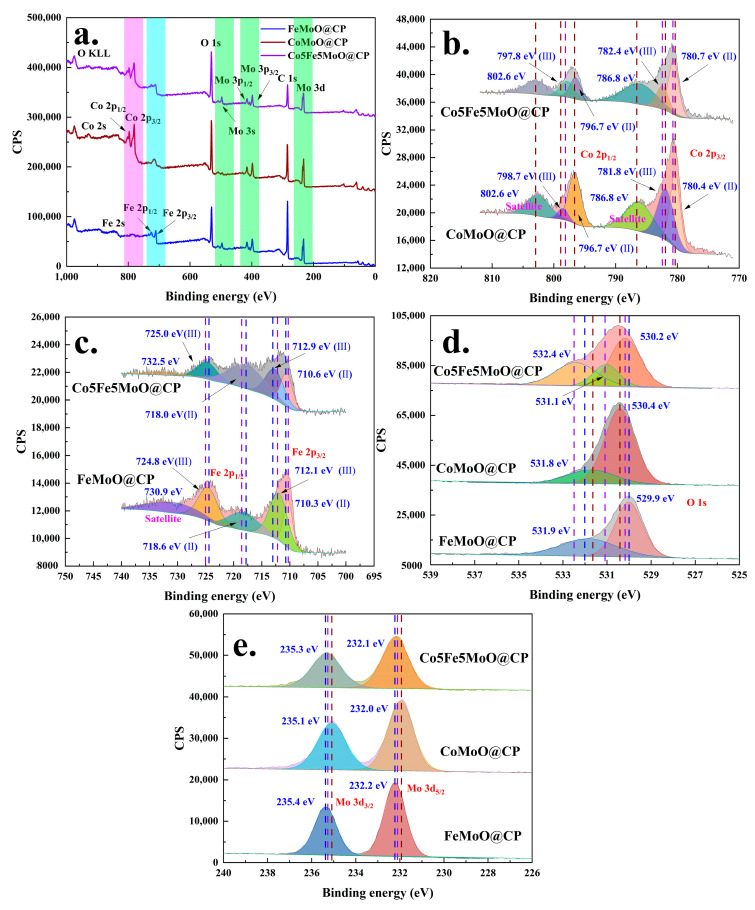
The XPS analysis results of CoMoO@CP, FeMoO@CP, and Co5Fe5MoO@CP materials: full spectrum scanning (**a**); high resolution XPS of Co2p (**b**); Fe 2p (**c**); O 1s (**d**); and Mo 3d (**e**).

XPS was a valuable technique for elucidating the surface elemental composition of materials. Figure 4 and Figure S19 presented the XPS analysis results for various composite materials, including CoMoO@CP, FeMoO@CP, Co5Fe5MoO@CP, and other CoxFe10-xMoO materials. The full spectrum scanning results (Figure 4a) revealed that for the FeMoO@CP material, XPS peaks corresponding to iron (Fe 2s, Fe 2p_1/2_, and Fe 2p_3/2_), molybdenum (Mo 3s, Mo 3p_1/2_, Mo 3p_3/2_, and Mo 3d), oxygen (O 1s), and carbon (C 1s) were observable. In contrast, the CoMoO@CP material primarily showed XPS peaks for cobalt (Co 2s, Co 2p_1/2_, and Co 2p_3/2_), along with molybdenum, oxygen, and carbon elements. The carbon peak (C 1s) presented in both materials was attributed to the carbon paper fibers within the composite material. For the Co5Fe5MoO@CP and other CoxFe10-xMoO composite materials that contained both cobalt and iron elements, XPS analysis showed peaks related to both two elements (iron and cobalt) in addition to molybdenum, oxygen, and carbon. Moreover, as the ratio of iron to cobalt varied, the intensity of the Co 2p and Fe 2p peaks adjusted accordingly, while the peaks for molybdenum, oxygen, and carbon remained relatively stable (see Figure S19).

Furthermore, Figure 4b presented the high-resolution XPS analysis results for the Co 2p region. The analysis data revealed that the peak shapes and positions for Co 2p in CoMoO@CP and Co5Fe5MoO@CP materials were similar. The Co 2p peaks were resolved into two components: Co 2p_1/2_ and Co 2p_3/2_. Then, each component could split into two peaks representing +2 and +3 valence, accompanied by satellite peaks. These peak positions and shapes indicated that cobalt in these composites predominantly existed in the +2 to +3 valence [34]. In contrast, the Fe 2p region, as depicted in Figure 4c, exhibited multiple peaks due to the variable valence states of iron. For the FeMoO@CP material, the XPS spectrum revealed peaks at 710.3 eV (Fe (Ⅱ) 2p_3/2_), 712.1 eV (Fe (Ⅲ) 2p_3/2_), 718.6 eV (Fe (Ⅱ) 2p_12_), and 724.8 eV (Fe (Ⅲ) 2p_1/2_) and a weak satellite peak at 730.9 eV. These results indicated the simultaneous presence of both divalent and trivalent iron in the FeMoO@CP material [35,36]. Similar peak shapes were observed in the Co5Fe5MoO@CP composite, although some peak positions and intensities were altered. Specifically, for the Co5Fe5MoO@CP material, the Fe 2p peaks were located at 710.3 eV (Fe (Ⅱ) 2p_3/2_), 712.1 eV (Fe (Ⅲ) 2p_3/2_), 718.6 eV (Fe (Ⅱ) 2p_12_), 724.8 eV (Fe (Ⅲ) 2p_1/2_), and 730.9 eV (satellite), with an enhanced intensity for Fe (Ⅱ) 2p_3/2_ and Fe (Ⅱ) 2p_1/2_. The observed changes indicated that, compared to pure iron molybdate, the valence state of iron (specifically, the relative proportions of +2 and +3 valence) had been altered in the dual-element molybdate materials. In addition, for the FeMoO@CP and CoMoO@CP materials, the O 1s peak (Figure 4d) could be deconvoluted into two components located approximately at 530 eV and 531 eV. These peaks corresponded to Mo-O and Co/Fe-O bonds in the single-element molybdates. In the case of the dual-element Co5Fe5MoO@CP composite, the O 1s peak was further resolved into three distinct peaks at around 530 eV, 531 eV, and 532 eV, reflecting the presence of Mo-O, Co-O, and Fe-O bonds, respectively. Moreover, for the molybdenum element, Figure 4e presented the high-resolution XPS analysis of the Mo 3d peaks. The data revealed that the Mo 3d peak in all materials could be deconvoluted into two components located near 232 eV and 235 eV. The minimal variation observed suggested that the oxidation state of molybdenum remained largely unchanged across all molybdate materials [37,38]. Overall, the XPS analysis confirmed the presence of cobalt, iron, molybdenum, and oxygen on the surface of the Co5Fe5MoO@CP composite, which was consistent with the findings from the SEM analysis. Given that cobalt and iron were considered potential active catalytic sites, their presence in these compositions was likely to contribute positively to the electrocatalytic performance of the material.

**Figure 5:**
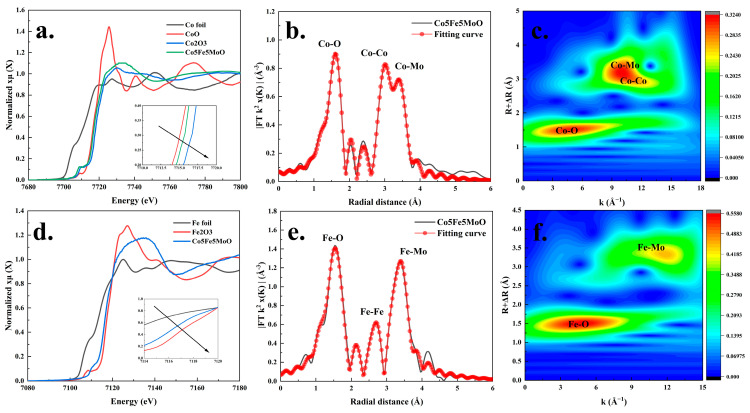
The Co K-edge XAFS analysis results of Co foil, CoO, Co_2_O_3_, and Co5Fe5MoO materials (**a**), the corresponding Fourier-transform and theoretical structure fitting spectra of Co for Co5Fe5MoO material (**b**), and the wavelet transform Co K-edge of Co5Fe5MoO material (**c**). The Fe K-edge XANES analysis results of Fe foil, Fe_2_O_3_, and Co5Fe5MoO materials (**d**), the corresponding Fourier-transform and theoretical structure fitting spectra of Fe for Co5Fe5MoO material (**e**), and the wavelet transform Co K-edge of Co5Fe5MoO material (**f**).

Next, the structure and elemental composition of the Co5Fe5MoO composite material were extensively investigated through X-ray Absorption Fine Structure (XAFS) technology, with the results presented in Figure 5. In Figure 5a, the Co K-edge XAFS analysis results (E-space) for Co foil, CoO, Co_2_O_3_, and Co5Fe5MoO materials were shown. Notably, the edge position of the Co5Fe5MoO material was significantly distanced from that of Co foil and laid between those of CoO and Co_2_O_3_. This observation suggested that the oxidation state of cobalt in the Co5Fe5MoO material was between +2 and +3 [39,40], aligning with the findings from the XPS analysis. The raw XAFS data were subsequently converted into a k^2^-weighted k-wave vector space, with results displayed in Figure S20a. Additionally, a Fourier transform was employed to analyze the weighted k-space data, fitting the results using the theoretical structures illustrated in Figure S20c and Figure 5b. As seen in Figure 5b, the primary bonds in the Co5Fe5MoO were located at 1.60, 2.98, and 3.39 Å, which corresponded closely to the theoretical bond lengths for Co-O (1.97 Å), Co-Co (3.27 Å), and Co-Mo (3.56 Å). The observed slight differences of approximately 0.3–0.4 Å between the theoretical and XAFS measured data were deemed reasonable.

Meanwhile, the satisfactory fitting results in Figure 5b and Figure S20c indicated that the theoretical model (Figure S1) aligned with the atomic structure of actual Co5Fe5MoO materials. The k^2^-weighted k-wave vector space data underwent a wavelet transform, with results shown in Figure 5c. In Figure 5c, the values along the vertical axis (R + ΔR) for the darkest area (tangerine) ranged between 1.34–1.68 Å and 2.85–3.61 Å, encompassing the Co-O, Co-Co, and Co-Mo bonds observed in the Fourier-transform results (Figure 5b). A similar analytical process was then applied to the Fe K-edge, with results displayed in Figure 5d–f. As seen in Figure 5d, the Fe near the absorption edge of the Co5Fe5MoO material fell between Fe foil and Fe_2_O_3_, leaning closer to Fe_2_O_3_, implying that the iron in the Co5Fe5MoO material also existed in an oxidation state between +2 and +3 [41,42]. Analyzing the Fourier-transform results (Figure 5e) of the k^2^-weighted data (Figure S20b) revealed that the primary chemical bonds associated with iron in the Co5Fe5MoO material were located at 1.56, 2.72, and 3.41 Å, which closely matched the actual bond lengths of Fe-O (1.92 Å), Fe-Fe (3.15 Å), and Fe-Mo (3.69 Å) in the theoretical structure. Similar to cobalt, the fitting results for iron (Figure 5e and Figure S20d) were also deemed acceptable. Furthermore, Figure 5f indicated that the vertical axis values (R + ΔR) for the darkest regions (tangerine and dark yellow) are primarily between 1.31–1.67 Å and 3.19–3.53 Å, encompassing the Fe-O and Co-Mo bonds from the Fourier-transform results (Figure 5e). Overall, XAFS analysis had determined the valence states and main bonds of both cobalt and iron, confirming the consistency between the theoretical model structure and the atomic structure of the Co5Fe5MoO material.

### 3.4. Electrochemical Catalytic Performance

**Figure 6:**
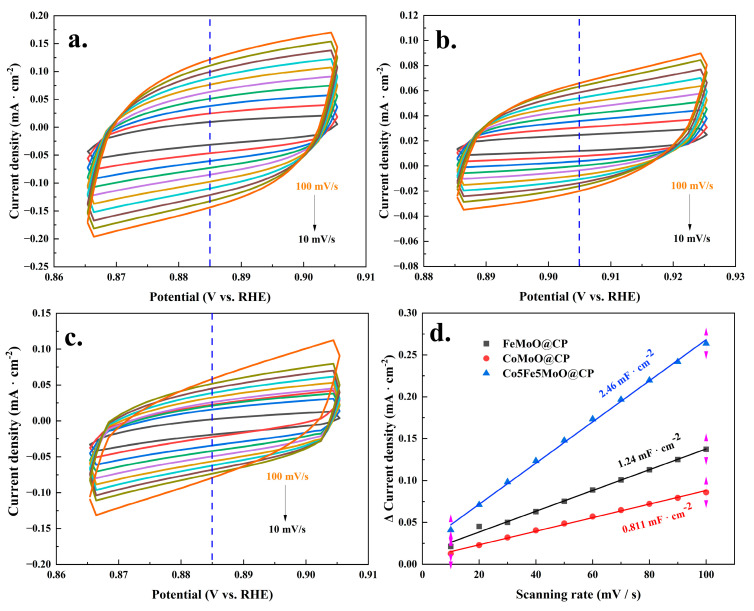
The CV curves of non-Faraday region (different scanning speeds): ((**a**): Co5Fe5MoO@CP, (**b**): CoMoO@CP, and (**c**): FeMoO@CP); plots of capacitive current density vs. different scan rate for C_dl_ (**d**).

The electrochemical active surface area (ECSA) was a critical parameter for assessing the catalytic activity of materials, and it could be derived from the cyclic voltammetry (CV) curves in the non-Faradic regions (no obvious oxidation–reduction reaction occurred). Figure 6 presented the results of the ECSA analysis. Figure 6a–c illustrated that, at different scanning speeds, the Co5Fe5MoO@CP system exhibited higher charge–discharge currents compared to CoMoO@CP, FeMoO@CP, and other cobalt/iron ratio composites (see Figure S21). The results indicated that the Co5Fe5MoO@CP composite exhibited a significantly higher density of electroactive sites, which played a pivotal role in enhancing its electrocatalytic performance by facilitating more efficient electron transfer and providing a larger place for catalytic reactions. Additionally, the plots of capacitive current density versus the scan rate, shown in Figure 6d and Figure S22, presented that the Co5Fe5MoO@CP composite achieved the highest capacitance value (2.46 mF·cm^−2^). This high capacitance further contributed to the improved electrochemical activity of the Co5Fe5MoO@CP composite [43].

**Figure 7:**
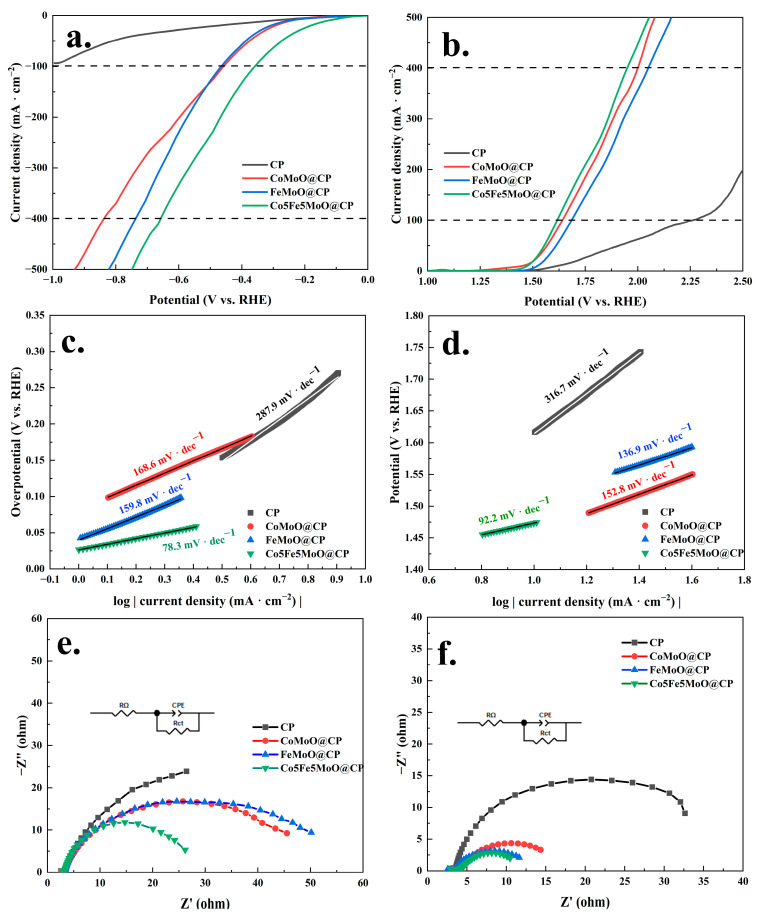
The HER IR-corrected LSV polarization curves (**a**), Tafel plots (**c**), and EIS spectra (**e**) of carbon paper, FeMoO@CP, CoMoO@CP, and Co5Fe5MoO@CP systems. The OER IR-corrected LSV polarization curves (**b**), Tafel plots (**d**), and EIS spectra (**f**) of carbon paper, FeMoO@CP, CoMoO@CP, and Co5Fe5MoO@CP systems.

Furthermore, linear sweep voltammetry (LSV) and subsequent data analysis were utilized to evaluate the HER and OER activities of materials, with the results depicted in Figure 7 and Figures S23–S26. As shown in Figure 7a, the HER overpotential for the Co5Fe5MoO@CP material was 123.6 mV at 10 mA·cm^−2^ (see Figure S23), 353.2 mV at 100 mA·cm^−2^, and 658.0 mV at 400 mA·cm^−2^. These values were significantly lower compared to those of single-element molybdate composites (FeMoO@CP and CoMoO@CP) and the original carbon cloth. Furthermore, when compared to dual-element molybdates with other cobalt/iron ratios (see Figure S24), the Co5Fe5MoO@CP composite demonstrated the lowest HER overpotential. The subsequent analysis of the steady-state region of the linear sweep voltammetry curves provided the Tafel slopes for the hydrogen evolution reaction, as illustrated in Figure 7c and Figure S25. The data indicated that, among single-element and dual-element molybdate systems (with varying cobalt/iron ratios), the Co5Fe5MoO@CP system exhibited the smallest Tafel slope value of 78.3 mV·dec^−1^. This suggested that the Co5Fe5MoO@CP system required the least potential increase to achieve a tenfold increase in reaction current density, thereby making the reaction process the most economical. Based on the Tafel slope values and the hydrogen evolution mechanism under alkaline conditions, it could be inferred that the Co5Fe5MoO@CP system followed the Volmer–Heyrovsky reaction pathway [44,45]. Moreover, electrochemical impedance spectroscopy (EIS) was then employed to assess the internal resistance and charge transfer resistance during the reaction process, with results presented in Figure 7e and Figure S26. The analysis revealed that, despite similar internal resistance values (R_Ω_: about 2.65 Ω) across all four systems due to comparable reaction setups, there were notable differences in charge transfer resistance (R_ct_). Specifically, the Co5Fe5MoO@CP system demonstrated the lowest charge transfer resistance of 11.2 Ω among all the materials, implying that its electronic transmission efficiency was the highest during the HER process [46,47].

**Figure 8:**
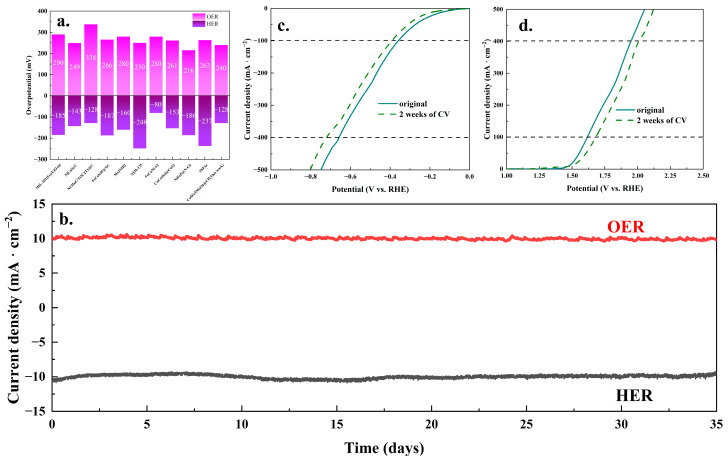
The recent literature results about HER and OER overpotential (**a**); the HER and OER i-t test of Co5Fe5MoO@CP material for 35 days (**b**); the HER and OER LSV polarization curves before and after the stability test of 2 weeks of CV cycles (**c**,**d**).

Continuously, Figure 7b,d,f and Figures S27–S30 presented the results of the electrocatalytic analysis for OER. The data revealed that, akin to the HER performance, the Co5Fe5MoO@CP system demonstrated the lowest OER overpotentials (245 mV at 10 mA·cm^−2^, 386 mV at 100 mA·cm^−2^, and 719 mV at 400 mA·cm^−2^), the smallest Tafel slope (92.2 mV·dec^−1^), and the lowest charge transfer resistance (4 Ω) among all the catalytic systems. Furthermore, Table S1 and Figure 8a compared the recent results of hydrogen and oxygen evolution for various electrocatalytic materials. Based on a comprehensive analysis of HER and OER overpotentials and Tafel slopes, the material studied in this research was positioned at an upper-middle performance level relative to other reported electrocatalysts. In addition, Figure S31 compared the electrocatalytic hydrogen and oxygen evolution results of Co5Fe5MoO@CP composite with commercial catalysts (Pt/C and RuO_2_). From Figure S31a,b, it was seen that for the hydrogen evolution reaction, the overpotential and Tafel slope of commercial Pt/C catalysts were lower at low current densities (below about 160 mA·cm^−2^), while the overpotential of Co5Fe5MoO@CP in this study was even lower at high current densities (over about 160 mA·cm^−2^). This indicated that the Co5Fe5MoO@CP composite obtained an advantage in the hydrogen evolution reaction at high current densities. Then, during the oxygen evolution reaction, compared with commercial RuO_2_ catalysts, Co5Fe5MoO@CP exhibited a lower OER overpotential and Tafel slope when the current density exceeded about only 50 mA·cm^−2^. Based on the combined results of HER and OER, it was seen that as the current density increased, the catalytic advantage of Co5Fe5MoO@CP in the electrocatalysis reaction process became increasingly apparent. Considering the high prices of Pt and Ru as noble metals, the Co5Fe5MoO@CP composite in this study displayed favorable competitiveness in applications. Overall, the findings suggested that an effective electrocatalytic material for both hydrogen and oxygen evolution could be achieved by anchoring metal molybdate on carbon paper. Specifically, the cobalt-to-iron ratio significantly impacted the electrocatalytic performance, with the 5:5 ratio exhibiting the most optimal catalytic properties.

**Figure 9:**
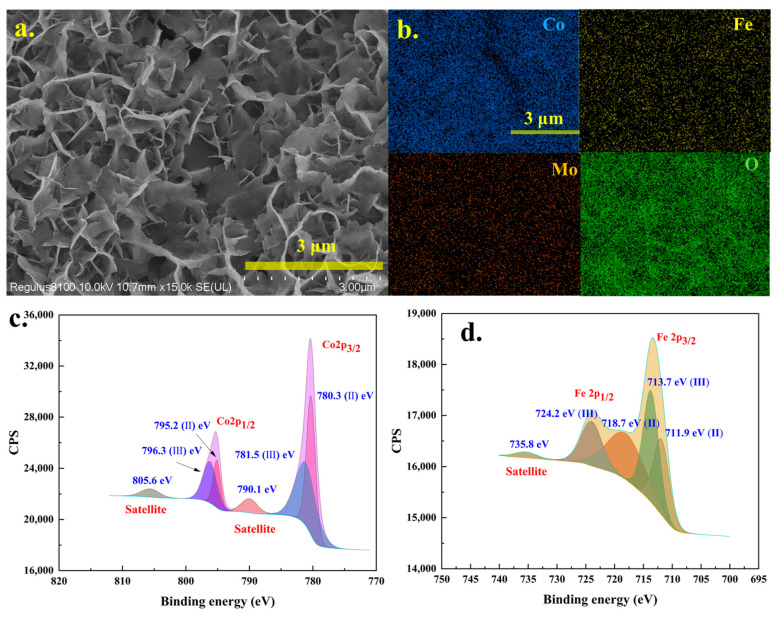
The SEM analysis results (**a**) and the EDX mapping analysis results (**b**) of Co5Fe5MoO@CP composite sequentially after HER and OER stability test; the high-resolution XPS analysis results of Co2p (**c**) and Fe 2p (**d**) of Co5Fe5MoO@CP composite sequentially after HER and OER stability test.

The stability of catalytic performance was crucial for the efficacy of catalytic materials. Figure 8b–d presented the results of the electrocatalytic stability tests for the Co5Fe5MoO@CP composite. As illustrated in Figure 8b, the current densities for both hydrogen and oxygen evolution reactions remained at 10 mA·cm^−2^ for 35 days at the appropriate potentials. Figure 8c further demonstrated that, after 2 weeks of CV cycles, the overpotential for the HER increased to 169.6 mV at 10 mA·cm^−2^, 388.9 mV at 100 mA·cm^−2^, and 717.9 mV at 400 mA·cm^−2^. Similarly, the overpotential for the OER increased to 261 mV at 10 mA·cm^−2^, 451 mV at 100 mA·cm^−2^, and 770 mV at 400 mA·cm^−2^ after 3000 CV cycles. These results indicated that the Co5Fe5MoO@CP material demonstrated satisfactory stability in catalytic performance. In addition to catalytic activity, the stability of the structure and elemental composition was also critical to catalytic materials. Figure 9 showed the SEM and XPS analysis results of the Co5Fe5MoO@CP composite after stability testing for both HER and OER. The SEM images in Figure 9a,b revealed that the material retained its nanoflower morphology and continued to contain cobalt, iron, molybdenum, and oxygen elements following the stability tests. Comparing the composition ratios of main elements before and after the stability reaction (Table 1), it can be seen that after a long period of hydrogen and oxygen evolution reactions, the surface elements of Co5Fe5MoO@CP composite were stable with little change in element ratios. High-resolution XPS analysis (Figure 9c,d) revealed that the peak positions and shapes of the cobalt and iron elements (key catalytic sites) remained relatively stable. This stability indicated that the valence states of cobalt and iron were preserved throughout the catalytic process. The results demonstrated that, following the HER and OER stability tests, the Co5Fe5MoO@CP composite maintained its excellent structural integrity and elemental composition, including the valence states of its constituent elements. This robustness in both catalytic performance and structural composition underscored the material’s suitability for subsequent practical applications.

**Figure 10:**
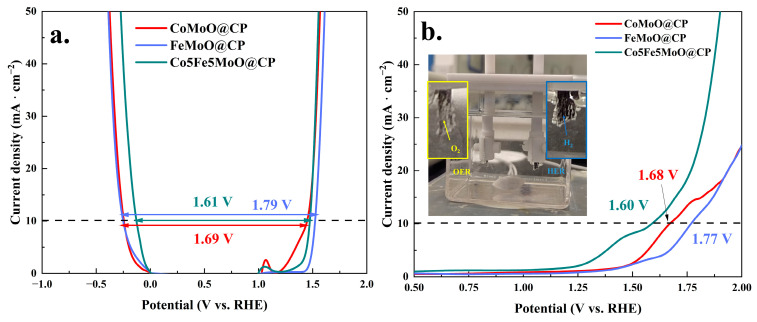
The HER and OER comprehensive analysis results: (**a**) the actual two electrode LSV overall water splitting analysis results and (**b**) the insert digital photo of overall water splitting.

The above research findings demonstrated that the Co5Fe5MoO@CP composite exhibited notable catalytic activity for both HER and OER. Figure 10a presented the combined results of HER and OER, revealing that in the Co5Fe5MoO@CP system, the potential difference between the two electrodes at a current density of 10 mA·cm^−2^ was 1.61 V. This value was significantly lower compared to that of the FeMoO@CP and CoMoO@CP systems. Similar findings were evident from the two-electrode overall water splitting reaction results depicted in Figure 10b. Specifically, a cell voltage of just 1.6 V, approximating the voltage of a commercial dry battery, sufficed to initiate the reaction and achieve a current density of 10 mA·cm^−2^ for the entire overall water splitting process. Driven by two commercial dry batteries, the overall water splitting reaction proceeded continuously and efficiently, as illustrated in Figure S31.

### 3.5. Industrial Application Potential

**Figure 11:**
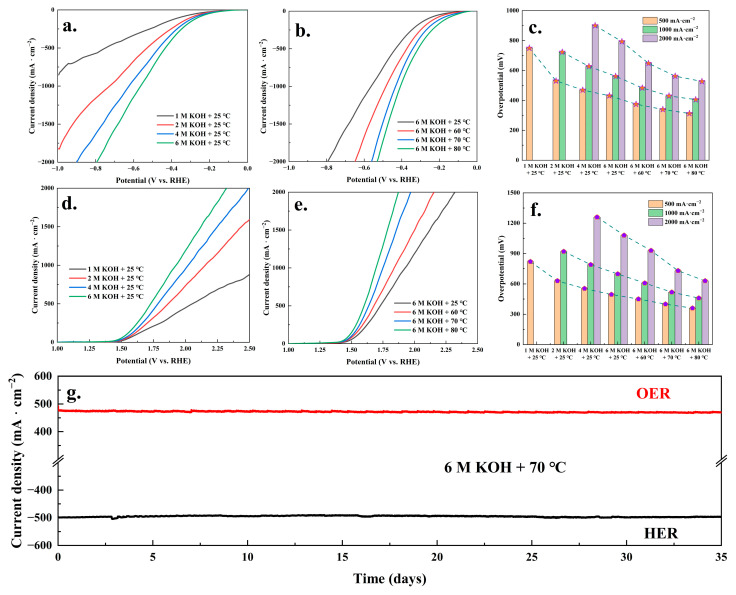
The HER IR-corrected LSV polarization curves of Co5Fe5MoO@CP system under different KOH concentrations and 25 °C (**a**) and different temperatures and 6 M KOH (**b**). The HER overpotential of catalytic systems at 500, 1000, and 2000 mA·cm^−2^ (**c**). The OER IR-corrected LSV polarization curves of Co5Fe5MoO@CP system under different KOH concentrations and 25 °C (**d**) and different temperatures and 6 M KOH (**e**). The OER overpotential of catalytic systems at 500, 1000, and 2000 mA·cm^−2^ (**f**). The HER and OER i-t test of Co5Fe5MoO@CP composite material for 35 days under 6 M KOH and 70 °C conditions (**g**).

From the above research, it could be seen that the Co5Fe5MoO@CP composite material displayed excellent HER and OER catalytic ability and stability. In addition, the potential for the industrial application of the Co5Fe5MoO@CP material was studied, and the results were shown in Figure 11. As we know, in the process of industrial electrocatalytic water splitting, the concentration of KOH electrolyte and operating temperature need to be increased. From the results in Figure 11a–c, it was seen that in the Co5Fe5MoO@CP system, an increase in KOH electrolyte (1 to 6 M) concentration and temperature (25 to 80 °C) was both beneficial for the HER reaction to proceed. For example, at 25 °C, when the current density was 500 mA·cm^−2^, the HER overpotential decreased from 749.8 mV of 1 M KOH to 430.9 of 6 M KOH. Then, under 6M KOH, when the current density was also 500 mA·cm^−2^, the HER overpotential continued to decrease from 430.9 mV under 25 °C to 312.4 mV under 80 °C. Furthermore, the increase in KOH concentration and temperature also had a significant promoting effect on the OER reaction. As presented in Figure 11d–f, at 25 °C, when the current density was 500 mA·cm^−2^, the OER overpotential decreased from 820.1 mV of 1 M KOH to 495.3 mV of 6 M KOH. Moreover, under 6M KOH, when the current density was also 500 mA·cm^−2^, the OER overpotential continued to decrease from 495.3 mV under 25 °C to 360.3 mV under 80 °C. Similar results had also been found in the studies of other researchers, which attributed to the increased temperature and alkaline concentration promoting the adsorption dynamics process of water molecules and hydroxide ions on the catalyst surface. Subsequently, Figure 11g showed the stability test (i-t) results of the Co5Fe5MoO@CP composite material at a high alkali concentration (6 M KOH) and temperature (70 °C). Figure 11g suggested that, similar to 1 M KOH and 25 °C system, the Co5Fe5MoO@CP material could also stably maintain the HER and OER current density of around 500 mA·cm^−2^ for 35 days under 6 M KOH and 70 °C conditions. Based on all the results of Figure 11, it could be known that under high alkaline concentration and temperature conditions, the Co5Fe5MoO@CP composite material could still maintain excellent HER and OER catalytic activity and stability, indicating its satisfactory potential for industrial applications.

### 3.6. Electrocatalytic Reaction Mechanism

Firstly, an effective electrocatalytic material was characterized by both rapid charge transfer capabilities and favorable theoretical catalytic properties. Characterization results indicated that Co5Fe5MoO nanosheets were successfully anchored onto the surface of carbon paper fibers via hydrothermal synthesis. Carbon paper, as a carbon-based material, was known for its excellent electron transfer properties, enabling efficient electron transport to the HER active sites on its surface and facilitating the transfer of electrons generated during the OER process. This setup provided a favorable environment for the overall water splitting reaction to occur effectively.

**Figure 12:**
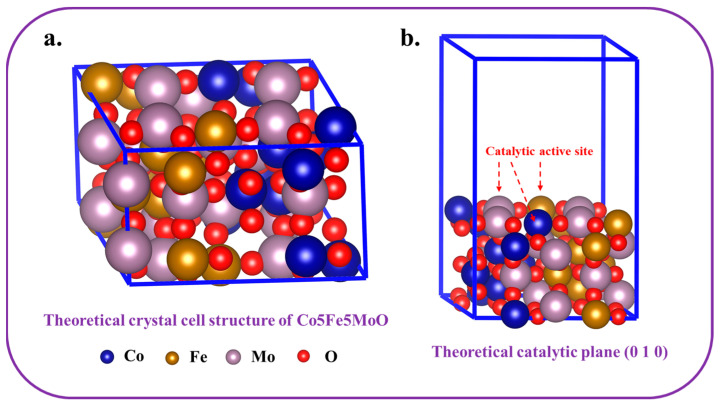
The theoretical crystal cell structure (**a**) and catalytic plane (0 1 0) of Co5Fe5MoO (**b**).

Additionally, theoretical catalytic abilities were assessed using DFT, a reliable method for theoretical analysis. Figure 12 showed the theoretical crystal cell structure and catalytic plane (0 1 0) of Co5Fe5MoO. From the theoretical crystal cell and surface catalytic structure, it was seen that iron, cobalt, and molybdenum atoms coexisted around the catalytic surface, and all these surface atoms might become catalytic active sites for HER and OER processes. With the aid of DFT, the optimal catalytic configuration and their Gibbs free energy could be obtained, thereby obtaining information about the active sites and Gibbs free energy change for each catalytic step.

As we know, in alkaline conditions, the theoretical hydrogen evolution process was typically divided into four key steps: (2) the adsorption of water molecules, (3) the catalytic decomposition of water, (4) the formation of hydroxide ions, and (5) hydrogen evolution [48].

(2) $$*(catalyst)+H_{2}O+e^{-}\to *-H_{2}O+e^{-}$$

(3) $$*-H_{2}O+e^{-}\to *-(H+OH)+e^{-}$$

(4) $$*-(H+OH)+e^{-}\to *-H+OH^{-}$$

(5) $$*-H+OH^{-}\to \frac{1}{2}H_{2}+*+OH^{-}$$

**Figure 13:**
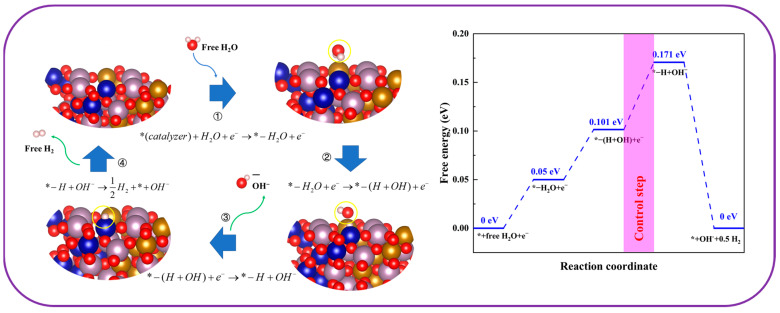
The theoretical optimal catalytic configuration (**left**) and change of Gibbs free energy along hydrogen evolution coordinate (**right**); * is the catalytic surface.

The effectiveness of the hydrogen evolution reaction was impacted by the changes in Gibbs free energy during each reaction phase. As depicted in Figure 13, the configurations optimized through calculations (Figure 12b) disclosed that during the HER, water molecules tended to adsorb primarily close to iron atoms. After the breakdown of water molecules, hydrogen atoms and hydroxyl groups gathered around cobalt and iron atoms; subsequently, the hydrogen atoms lingered close to the cobalt atoms following the release of hydroxide ions. The findings demonstrated that iron atoms acted as the principal active sites during the first adsorption of water molecules. After this initial phase, iron and cobalt worked in tandem to catalyze the decomposition of water, with iron assisting in hydroxyl group formation and cobalt facilitating hydrogen evolution. In conclusion, a remarkable synergistic effect was observed between iron and cobalt atoms on the Co5Fe5MoO surface, leading to improved efficiency of HER process. Theoretical calculations based on DFT revealed that the free energy changes for the four reaction steps on this catalyst were 0.05 eV, 0.051 eV, 0.07 eV, and −0.171 eV, respectively. The highest energy barrier was associated with the evolution of hydroxide ions, making it the rate-limiting step of the reaction.

(6) $$*(catalyst)+4OH^{-}\to *-OH+3OH^{-}+e^{-}$$

(7) $$*-OH+3OH^{-}+e^{-}\to *-O+H_{2}O+2OH^{-}+2e^{-}$$

(8) $$*-O+H_{2}O+2OH^{-}+2e^{-}\to *-OOH+H_{2}O+OH^{-}+3e^{-}$$

(9) $$*-OOH+H_{2}O+OH^{-}+3e^{-}\to *+O_{2}+2H_{2}O+4e^{-}$$

**Figure 14:**
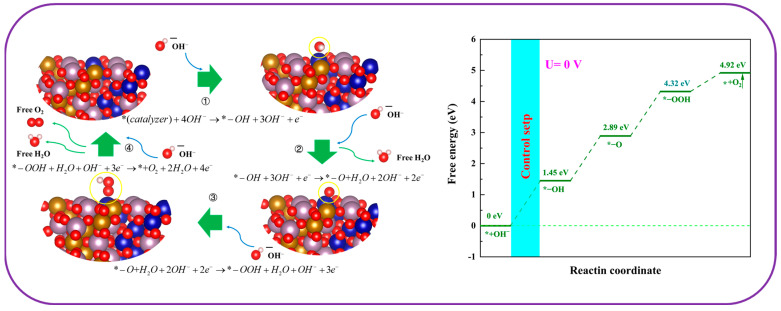
The theoretical optimal catalytic configuration (**left**) and change of Gibbs free energy along oxygen evolution coordinate (**right**). * is the catalytic surface.

The oxygen evolution reaction (OER), comparable to the hydrogen evolution reaction (HER), was structured into four distinct steps, as characterized by Equations (6) to (9) [44,49]. The DFT calculation findings for these steps were displayed in Figure 14. Regarding optimal configurations, hydroxyl groups, oxygen atoms, and OOH groups were primarily found in proximity to cobalt atoms. Consequently, in contrast to the HER process, the cobalt atoms played a unique role as the exclusive catalytic active sites for the OER. Additionally, unlike the HER process, the OER presented a notable free energy increase of 4.92 eV from the initial reactants to the end products. A substantial free energy barrier required that the OER took place in a progression of gradual steps, with each step being interconnected and limited by the total increase in free energy [50,51]. The free energy variations for each step in the OER were recorded at 1.45 eV, 1.44 eV, 1.43 eV, and 0.6 eV, respectively. The initial step, which represented the highest increase in free energy, was pinpointed as the rate-limiting step of the overall reaction. This step was essential as its energy demands influenced the feasibility of the subsequent steps.

**Figure 15:**
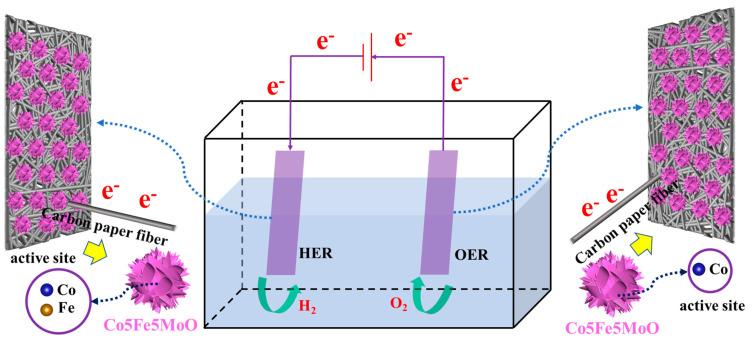
The summary of theoretical HER and OER catalytic mechanisms.

Based on the acknowledged features of carbon paper and the results from DFT analyses of catalytic surface processes (both HER and OER), the entire catalytic mechanism could be summarized as depicted in Figure 15. In the HER process, the carbon paper fibers played a crucial role in efficiently transferring electrons to the active sites of the Co5Fe5MoO composite located on the surface. The hydrogen evolution reaction was accomplished through four consecutive steps: water molecule adsorption, catalytic water decomposition, hydroxide ion formation, and hydrogen generation, facilitated by the synergistic actions of iron and cobalt atoms. Notably, the cobalt atom in Co5Fe5MoO functioned as a solitary active site during the oxygen evolution reaction, facilitating the sequential progression of the four-step OER process. Subsequently, the electrons produced throughout the reaction were efficiently shuttled away by the carbon paper fibers, thereby enabling the concurrent operation of both HER and OER reactions, with Fe and Co serving as active sites for HER and Co as the active site for OER.

## 4. Conclusions

Herein, cobalt/iron molybdate materials were successfully anchored onto the surface of carbon paper fibers via the hydrothermal method and obtained composite electrocatalysts (CoxFexMoO@CP). The morphology and catalytic efficiencies of composites were optimized by adjusting the ratio of cobalt to iron. Under the optimal mass proportion (Co/Fe = 5/5), the composite (Co5Fe5MoO@CP) displayed a distinctive nanoflower morphology, with 50–100 nm in thickness. The electrocatalytic findings revealed that, compared to the other composites with different proportions of cobalt and iron, Co5Fe5MoO@CP demonstrated the strongest electrocatalytic prowess for both HER and OER and enhanced the efficiencies of overall water splitting, which could be ascribed to a large number of active catalytic sites offered on the nanoflower surface. Notably, the overpotential for HER was 123.6 mV at a current density of 10 mA·cm^−2^, with a Tafel slope of 78.3 mV·dec^−1^. For OER, the overpotential and the Tafel slope was 245 mV and 92.2 mV·dec^−1^, respectively. Additionally, the Co5Fe5MoO@CP demonstrated consistent electrocatalytic performance over a span of 35 days and during 2 weeks of cyclic voltammetry tests. Even under conditions of elevated alkaline concentration and temperature, the Co5Fe5MoO@CP composite proved remarkable catalytic activity and stability for both HER and OER, highlighting its promising potential for industrial use. The overall water splitting reaction was efficiently achieved at a cell voltage of only 1.60 V. Furthermore, carbon paper, displaying excellent electron transfer potency, enabled efficient electron transport to the HER and OER active sites on the composite surface. DFT calculations further illustrated that the synergistic action of iron and cobalt atoms on the Co5Fe5MoO surface facilitated the efficient HER process, while cobalt atoms were identified as the primary catalytic sites for the OER process. This study offers a new approach to develop highly efficient electrocatalytic materials to greatly improve the overall water splitting process and has a promising potential for industrial application.

## Figures and Tables

**Table 1:** The XPS analysis results of surface element compositions of Co5Fe5MoO@CP composite sequentially after HER and OER stability test (atomic %).

Element	Compositions (Before Stability Test)	Compositions (After Stability Test)
Co	4.39	4.17
Fe	4.72	4.12
Mo	8.81	8.33
O	38.31	35.92
C	43.77	47.46
Total	100	100

## Data Availability

The original contributions presented in this study are included in the article/Supplementary Material. Further inquiries can be directed to the corresponding author.

## Supplementary Materials

The following supporting information can be downloaded at https://www.mdpi.com/article/10.3390/molecules30040844/s1. Figure S1: The theoretical cell configuration of Co5Fe5MoO from different visual angles; Figure S2: The theoretical catalytic model of Co5Fe5MoO; Figure S3: The theoretical catalytic model of *+H2O; Figure S4: The theoretical catalytic model of *+H; Figure S5: The theoretical catalytic model of *+(OH+H); Figure S6: The theoretical catalytic model of *+O; Figure S7: The theoretical catalytic model of *+OH; Figure S8: The theoretical catalytic model of *+OOH; Figure S9: The XRD analysis results of FeMoO, CoMoO, and CoxFe10-xMoO series materials; Figure S10: The SEM analysis results of Co5Fe5MoO@CP composite (high magnification); Figure S11: The SEM-EDX element mapping analysis results of Figure S10 (Co5Fe5MoO@CP composite); Figure S12: The SEM-EDX element mapping analysis results of Figure 3b (Co5Fe5MoO@CP composite); Figure S13: The SEM analysis results of FeMoO@CP material; Figure S14: The SEM-EDX element mapping analysis results of Figure S13 (FeMoO@CP material); Figure S15: The SEM analysis results of Co3Fe7MoO@CP material; Figure S16: The SEM-EDX element mapping analysis results of Figure S15 (Co3Fe7MoO@CP material); Figure S17: The SEM analysis results of CoMoO@CP material; Figure S18: The SEM-EDX element mapping analysis results of Figure S17 (CoMoO@CP material); Figure S19: The full spectrum scanning XPS analysis results of CoxFe10-xMoO@CP materials; Figure S20: The k2-weighted k-wave vector space data of Co5Fe5MoO material (a: Co K-edge and b: Fe K-edge); the comparison of actual and fitted curves of k-space data (c: Co K-edge and d: Fe K-edge); Figure S21: The CV curves of non-Faraday region (different scanning speeds) (a: Co2Fe8MoO@CP, b: Co3Fe7MoO@CP, c: Co4Fe6MoO@CP, d: Co6Fe4MoO@CP, e: Co7Fe3MoO@CP, and f: Co8Fe2MoO@CP); Figure S22: The plots of capacitive current density vs. different scan rates for Cdl; Figure S23: The HER IR-corrected LSV polarization curves (detail) of CoMoO@CP, FeMoO@CP, and Co5Fe5MoO@CP materials; Figure S24: The HER IR-corrected LSV polarization curves (a and b: detail) of CoxFe10-xMoO@CP materials; Figure S25: The HER Tafel plots of CoxFe10-xMoO@CP materials; Figure S26: The HER EIS spectra of CoxFe10-xMoO@CP materials; Figure S27: The OER IR-corrected LSV polarization curves (detail) of CoMoO@CP, FeMoO@CP, and Co5Fe5MoO@CP materials; Figure S28: The OER IR-corrected LSV polarization curves (a and b: detail) of CoxFe10-xMoO@CP materials; Figure S29: The OER Tafel plots of CoxFe10-xMoO@CP materials; Figure S30: The OER EIS spectra of CoxFe10-xMoO@CP materials; Figure S31: The comparison of hydrogen and oxygen evolution efficiency between Co5Fe5MoO@CP material and commercial Pt/C and RuO2 electrocatalysts (a and b: HER LSV and Tafel slope; c and d: OER LSV and Tafel slope); Table S1: The overall water splitting performance of recent electrocatalytic materials. References [52,53,54,55,56,57,58,59,60,61,62,63,64,65,66] are cited in the Supplementary Materials.
