# Supplementary material for: Synergistic Interactions Among Iron and Cobalt Atoms Within Bimetallic Molybdate@Carbon Paper Composite Create Bifunctional Nanoflower Electrocatalyst, Enhancing Efficiency for Overall Water Splitting in Alkaline Environment
Source: Molecules. 2025 Feb 12;30(4):844. doi: 10.3390/molecules30040844 (PMC11858107; doi:10.3390/molecules30040844)
Supplement: Supplementary file 1 [file molecules-30-00844-s001.zip › molecules-3467805-supplementary.pdf]

S1. Experiments and Methods

S1.1 Materials Characterization

To thoroughly evaluate the synthesized composite materials, a range of advanced analytical techniques was employed to characterize their various properties. Specifically, X-ray diffraction (XRD, Shimadzu XD-3A diffractometer, Shimadzu, Kyoto, Japan), scanning electron microscopy with energy dispersive spectroscopy (SEM-EDX, HITACHI Regulus 8100 HITACHI, Tokyo, Japan), transmission electron microscopy (TEM, JEOL JEM 2100F JEOL, Tokyo, Japan), and X-ray photoelectron spectroscopy (XPS, PHI 5000 VersaProbe ULVAC-PHI, Matsuzaki, Japan) were utilized. The characterization procedures are detailed as follows: (1) Morphological and elemental analysis: The morphological features and elemental composition of the materials were analyzed using a HITACHI Regulus 8100 scanning electron microscope and JEM-2100F transmission electron microscope. Both instruments enabled precise observation and assessment of the surface structure and distribution of elements within the materials. (2) Crystalline phase identification: XRD patterns were obtained using a Shimadzu XD-3A diffractometer, employing Cu-Kα radiation (λ = 1.54056 Å). This technique was instrumental in determining the crystalline phases present in the materials and assessing their mineral phase composition. (3) Surface chemistry and elemental composition: The surface chemistry and elemental composition were examined using a PHI 5000 VersaProbe XPS system. XPS analysis provided detailed information on the chemical states and bonding environments of the elements within the materials. By integrating these advanced characterization techniques, a comprehensive understanding of the structural, morphological, and chemical properties of the synthesized composite materials was achieved. This in-depth analysis facilitated insights into their electrocatalytic performance and potential applications.

The Co K-edge and Fe K-edge X-ray Absorption Fine Structure (XAFS) spectra were recorded in transmission mode and Lytle-fluorescence mode, respectively, at the BL14W1 beamline of the Shanghai Synchrotron Radiation Facility (SSRF). Data acquisition involved a fixed-exit Si (111) double-crystal monochromator, with energy calibration performed using metal foils. Additionally, XAFS spectra of standard samples, including Co foil, Fe foil, CoO, Co_2O_3, and Fe_2O_3, were collected in transmission mode. The resulting EXAFS data were processed using the ATHENA module of the IFEFFIT software package, adhering to standard procedures. Conversion of the k-wave vector space and Fourier-transform r-space data utilized k^2 weighting, and fitting of r-space and k-space data was conducted using the Artemis module based on theoretical models shown in Fig. S1. The wavelet transform employed the Morlet equation model.

S1.2 DFT Theoretical Calculation

In our research, the Vienna Ab initio Simulation Package (VASP) and spin-polarized Density Functional Theory (DFT) were utilized to investigate the mechanisms of HER and OER reaction processes [1]. The exchange-correlation potential was described using the Perdew–Burke–Ernzerhof (PBE) functional [2, 3], within the framework of the generalized gradient approximation (GGA) [4, 5]. The calculations employed a plane-wave cutoff energy of 350 eV and a K-point grid of 3×3×3. The energy convergence criterion was set at 10^{-4} eV. Prior to the detailed calculations, all theoretical structures were fully optimized. Fig. S1 illustrated the theoretical unit cell of Co5Fe5MoO. The theoretical cell structure of Co5Fe5MoO included 8 cobalt atoms, 8 iron atoms, 16 molybdenum atoms, and 64 oxygen atoms. The space group was P1, with cell parameters of 10.221 Å (a), 9.275 Å (b), 14.048 Å (c), 90.0 ° (α), 106.87 ° (β), and 90.0 ° (γ). Based on this cell structure, the surface catalytic models of Co5Fe5MoO were constructed, as depicted in Fig. S2. The catalytic crystal plane selected for analysis was the (010) plane. To mitigate periodic interactions, a vacuum layer of 15 Å was introduced into the slabs of the surface catalytic structure. Fig. S3 to Fig. S8 displayed the optimized catalytic structures for various adsorption states, including water molecule adsorption (*-H_2O), hydrogen atom adsorption (*-H), co-adsorption of hydrogen atom and hydroxyl group (*-(O+OH)), oxygen atom adsorption (*-O), hydroxyl adsorption (*-OH), and OOH group adsorption (*-OOH).

The Gibbs free energy for each catalytic structure was calculated using the following formula:

$$G = E_H + E_{ZPE} - TS \qquad (S1)$$

where E_H represented the total energy, E_{ZPE} was the zero-point vibrational energy, S denoted entropy, and T was the absolute temperature. All values were derived from DFT calculations, with the exception of the entropy values for small molecules such as H_2 and H_2O, which were obtained from standard thermodynamic tables.

S2. Figures

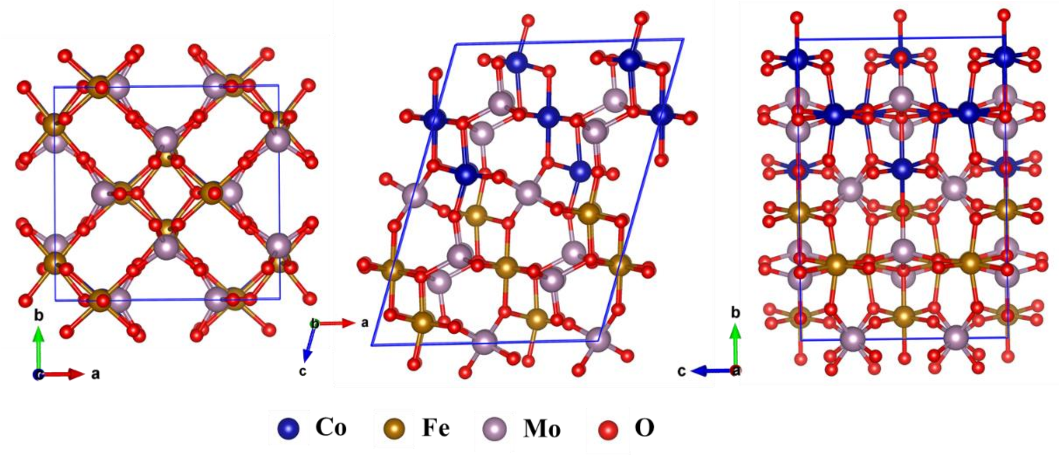

Fig. S1 The theoretical cell configuration of Co5Fe5MoO from different visual angles

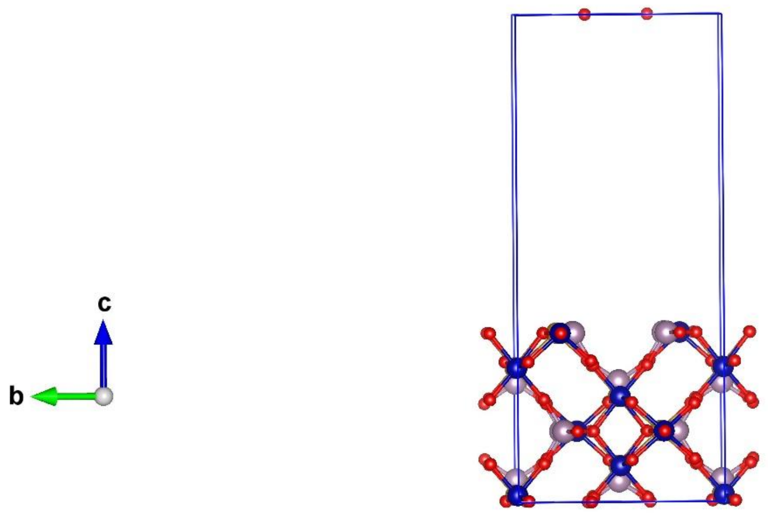

Fig. S2 The theoretical catalytic model of Co5Fe5MoO

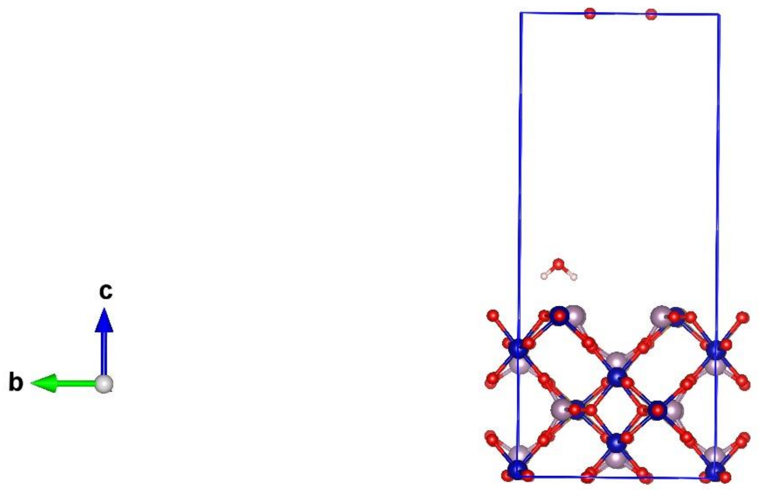

Fig. S3 The theoretical catalytic model of *+H_2O

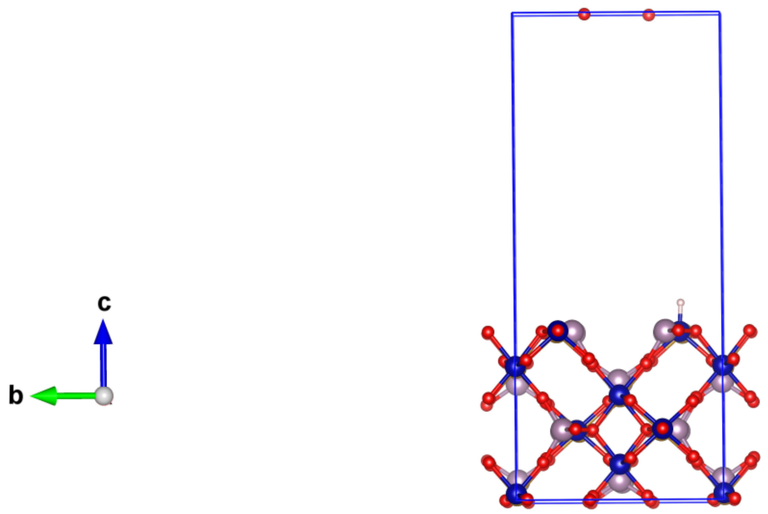

Fig. S4 The theoretical catalytic model of *+H

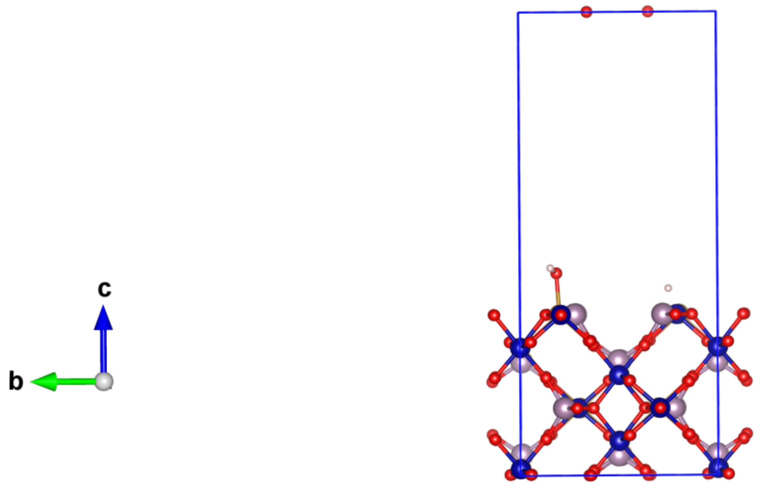

Fig. S5 The theoretical catalytic model of *+(OH+H)

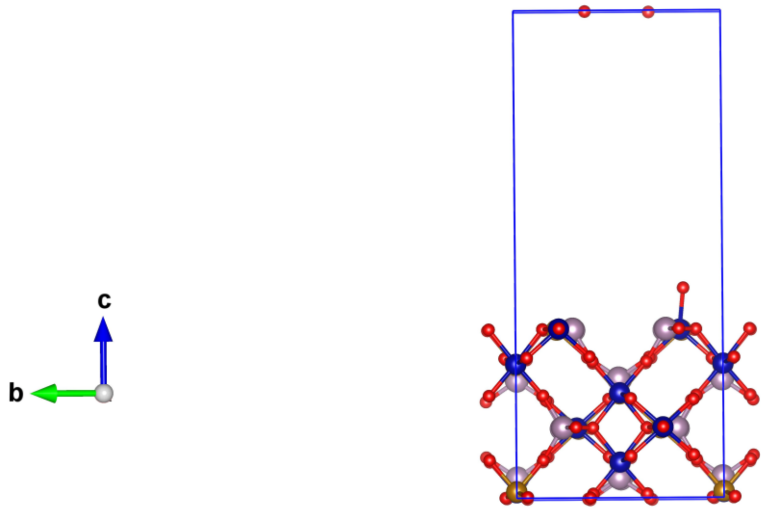

Fig. S6 The theoretical catalytic model of *+O

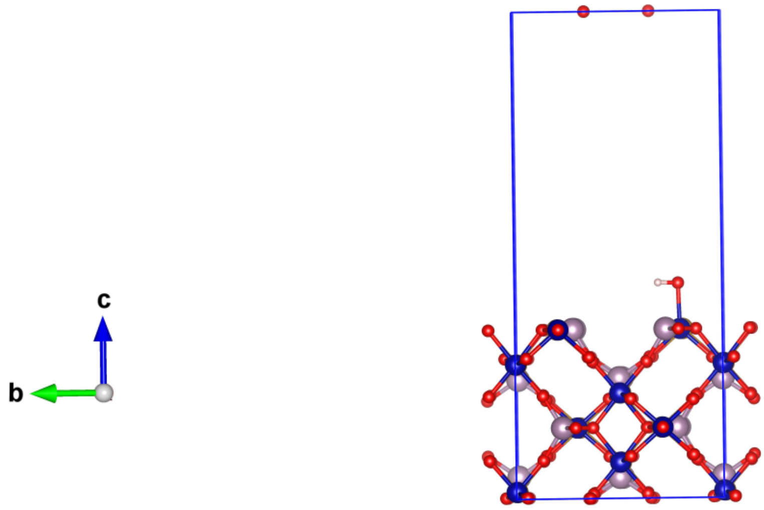

Fig. S7 The theoretical catalytic model of *+OH

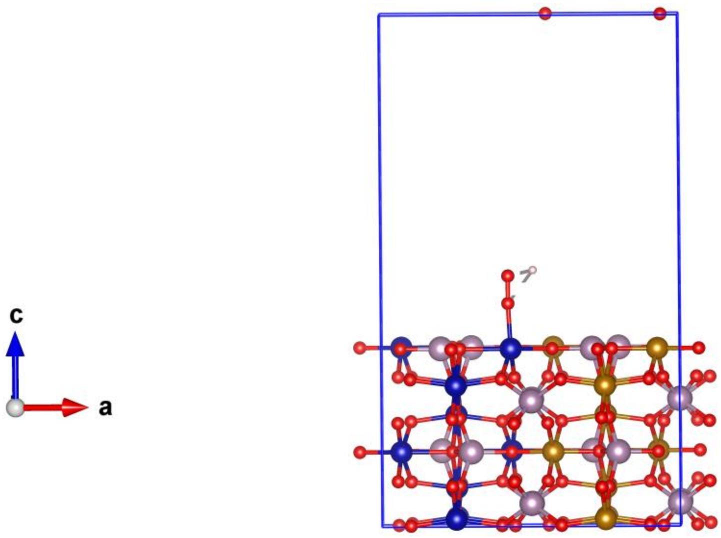

Fig. S8 The theoretical catalytic model of *+OOH

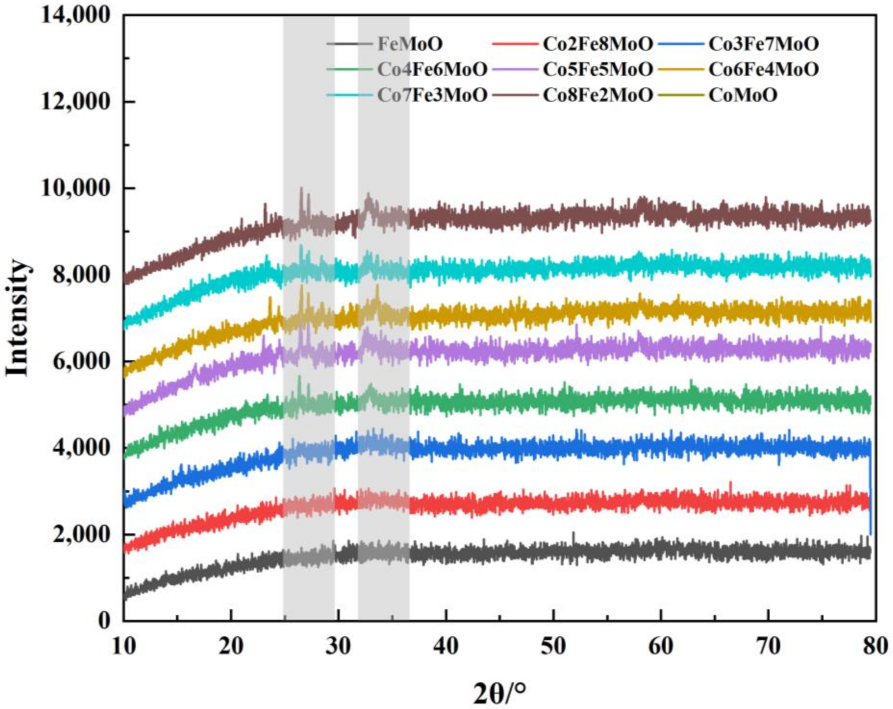

Fig. S9 The XRD analysis results of FeMoO, CoMoO, and CoxFe10-xMoO series materials

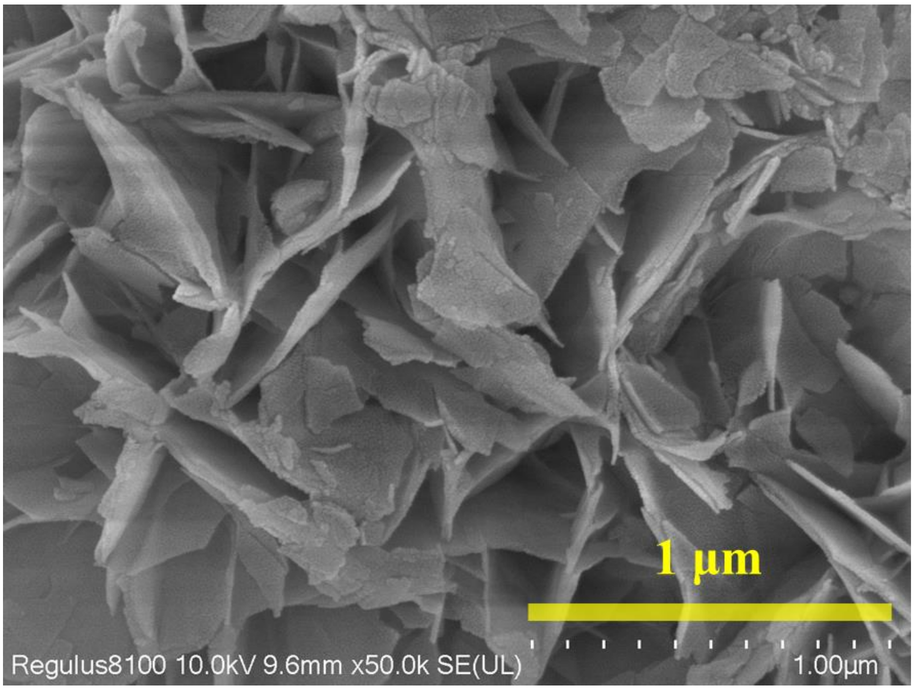

Fig. S10 The SEM analysis results of Co5Fe5MoO@CP composite (high magnification)

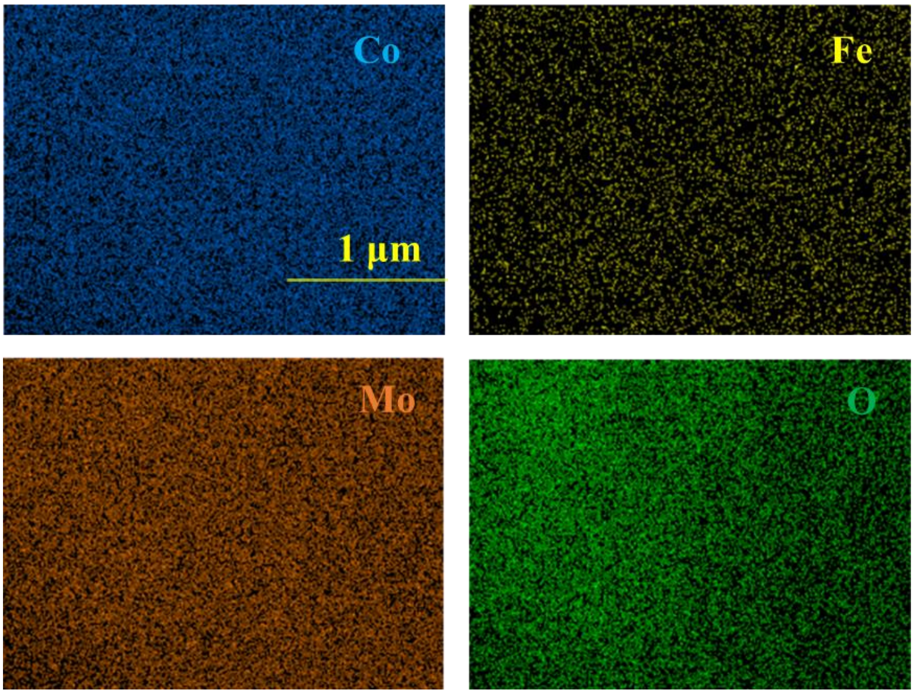

Fig. S11 The SEM-EDX element mapping analysis results of Fig. S10 (Co5Fe5MoO@CP composite)

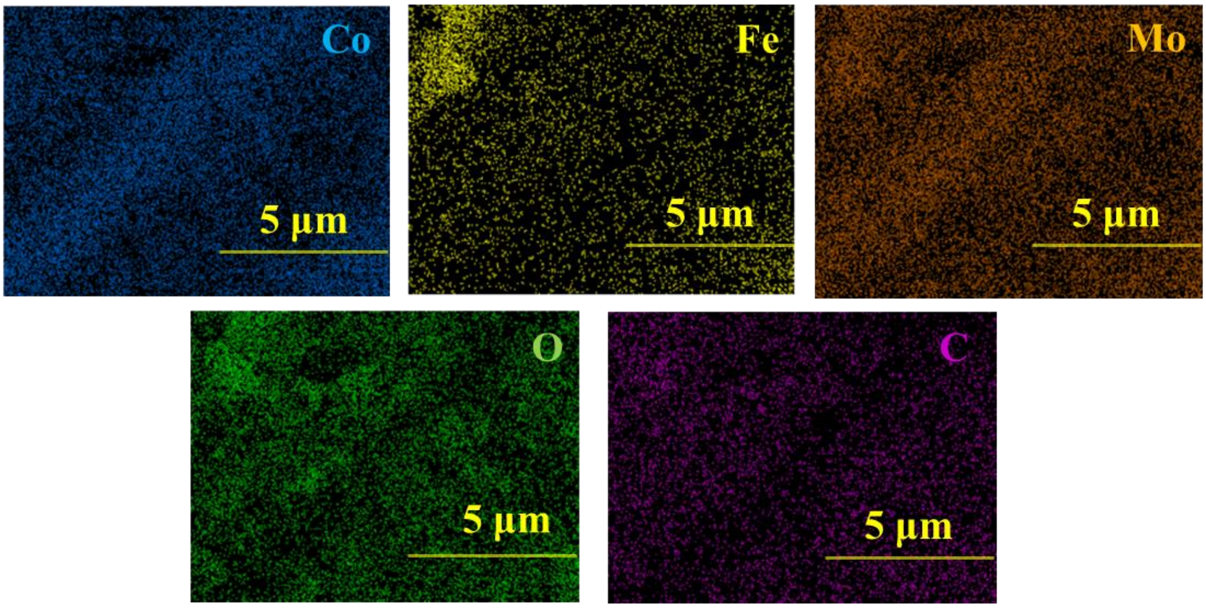

Fig. S12 The SEM-EDX element mapping analysis results of Fig.3b (Co5Fe5MoO@CP composite)

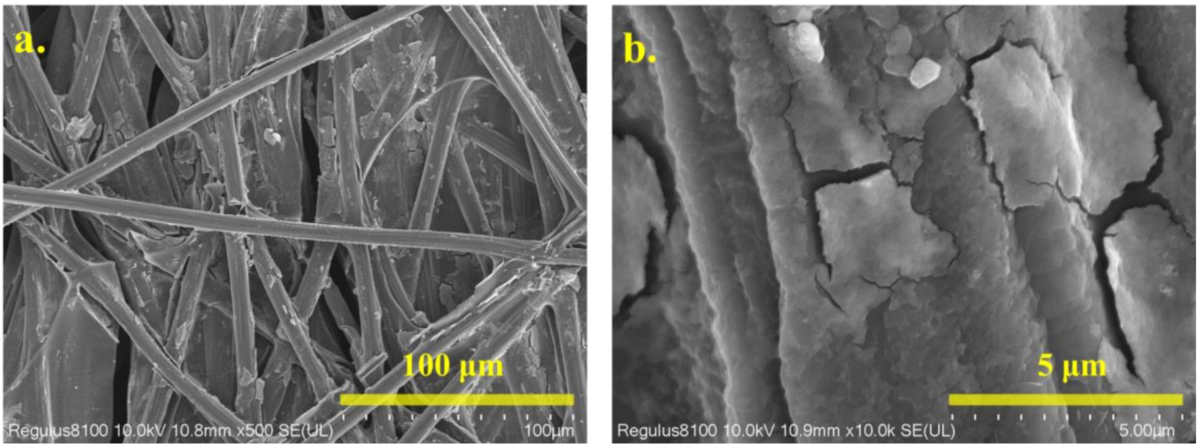

Fig. S13 The SEM analysis results of FeMoO@CP material

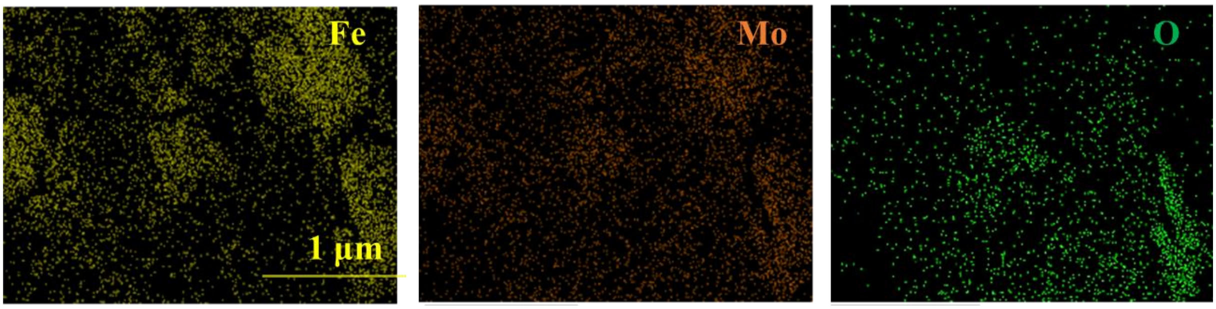

Fig. S14 The SEM-EDX element mapping analysis results of Fig. S13 (FeMoO@CP material)

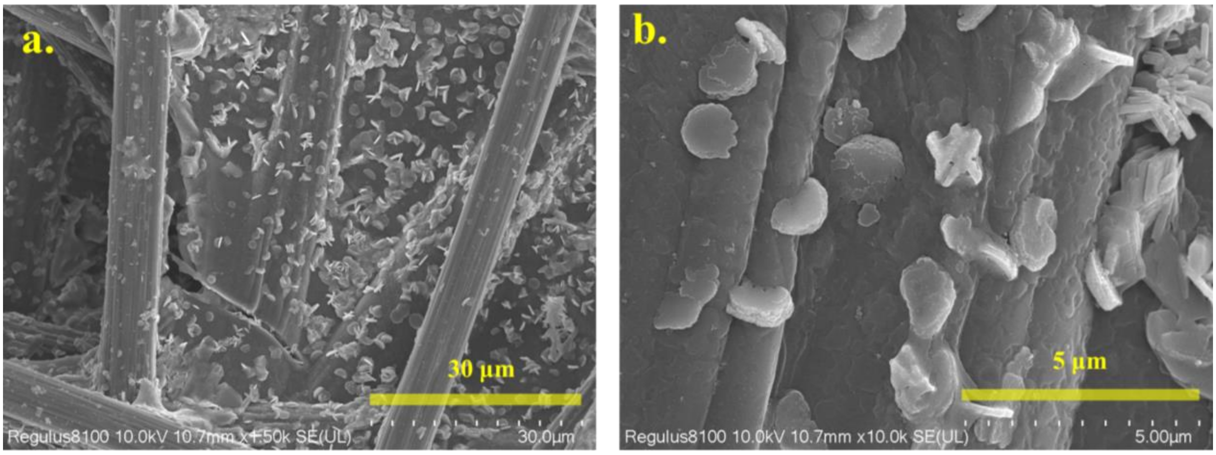

Fig. S15 The SEM analysis results of Co3Fe7MoO@CP material

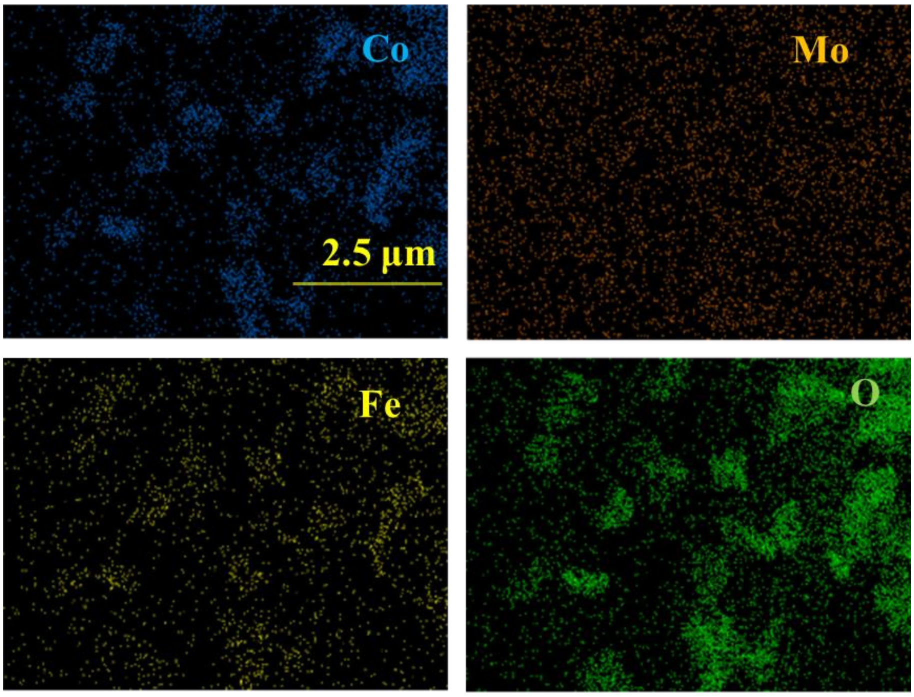

Fig. S16 The SEM-EDX element mapping analysis results of Fig. S15 (Co3Fe7MoO@CP material)

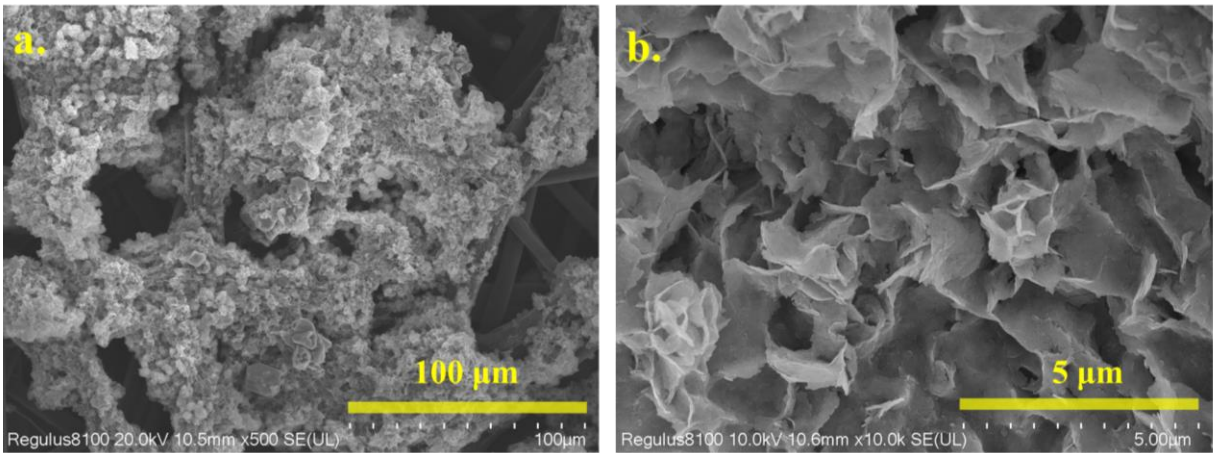

Fig. S17 The SEM analysis results of CoMoO@CP material

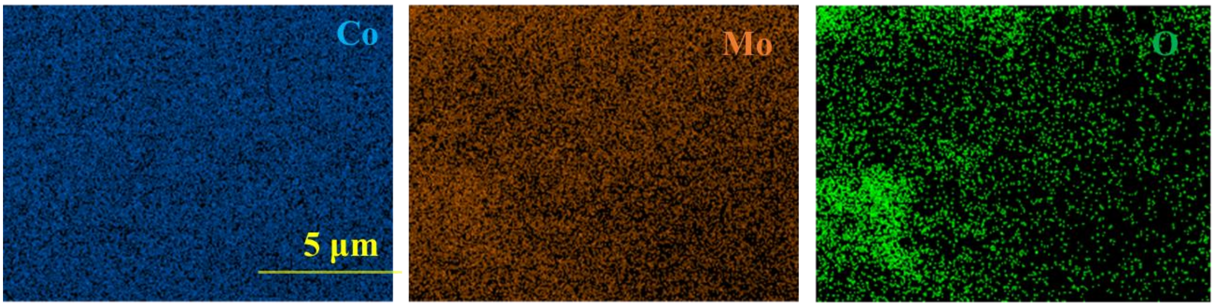

Fig. S18 The SEM-EDX element mapping analysis results of Fig. S17 (CoMoO@CP material)

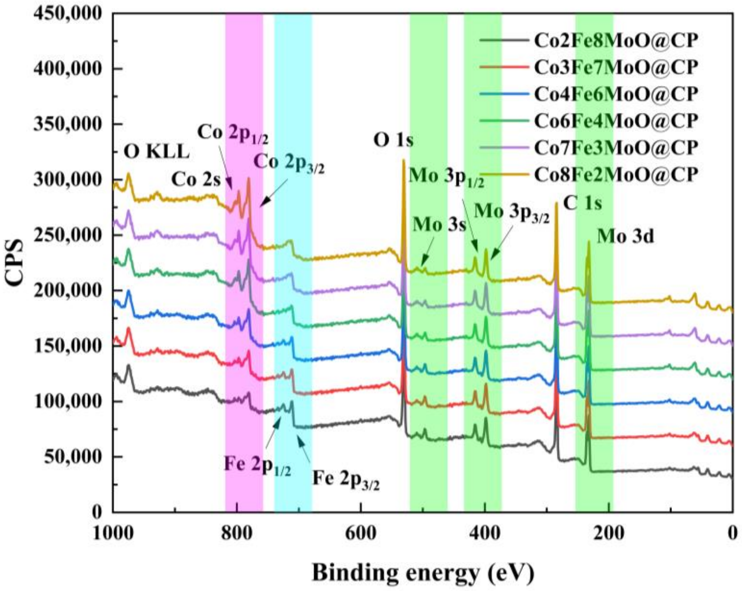

Fig. S19 The full spectrum scanning XPS analysis results of CoxFe10-xMoO@CP materials

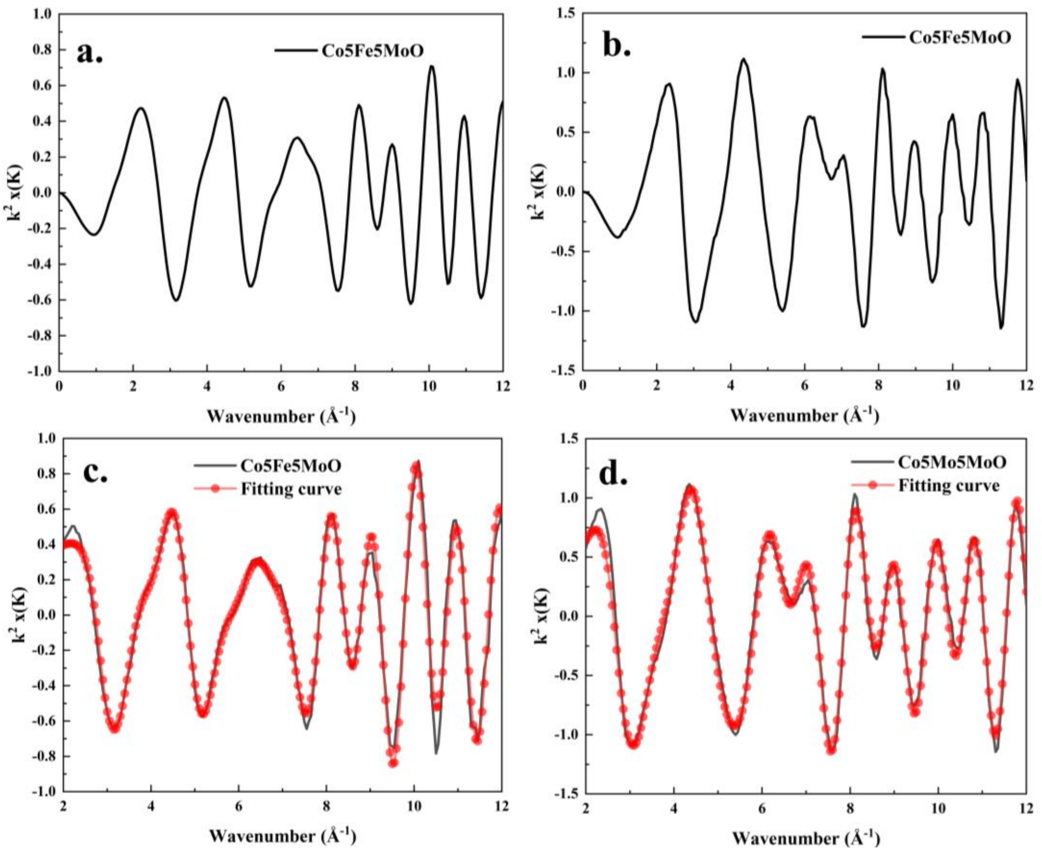

Fig. S20 The k^2-weighted k-wave vector space data of Co5Fe5MoO material (a: Co K-edge and b: Fe K-edge); the comparison of actual and fitted curves of k-space data (c: Co K-edge and d: Fe K-edge)

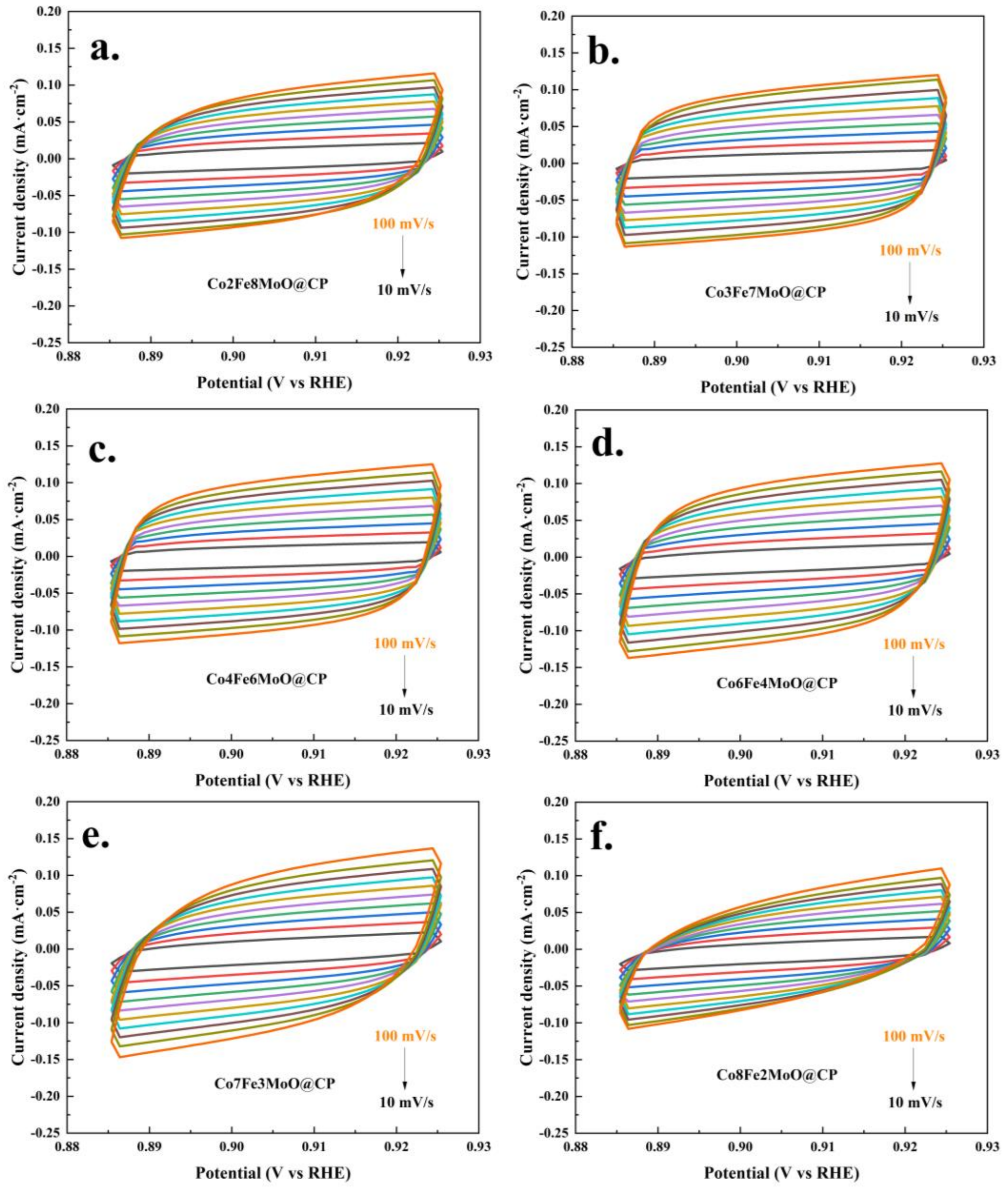

Fig. S21The CV curves of non-Faraday region (different scanning speeds) (a: Co2Fe8MoO@CP, b: Co3Fe7MoO@CP, c: Co4Fe6MoO@CP, d: Co6Fe4MoO@CP, e: Co7Fe3MoO@CP, and f: Co8Fe2MoO@CP)

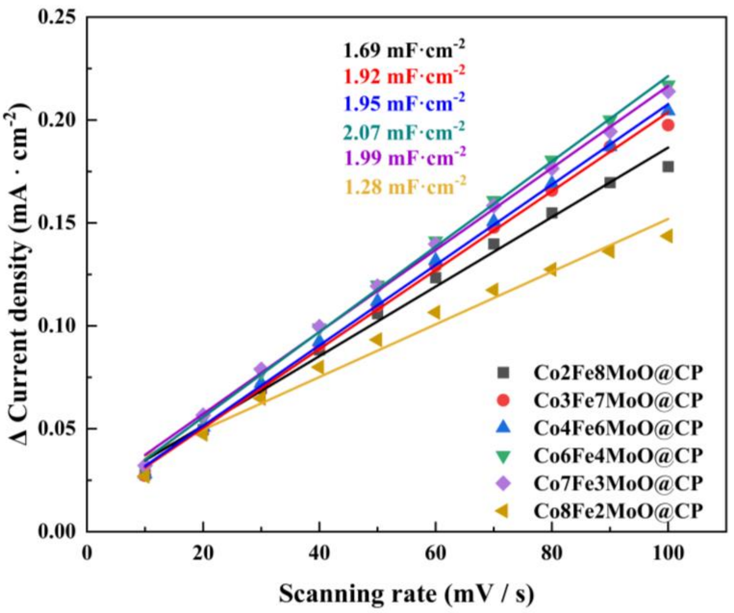

Fig. S22 The plots of capacitive current density vs. different scan rates for C_{dl}

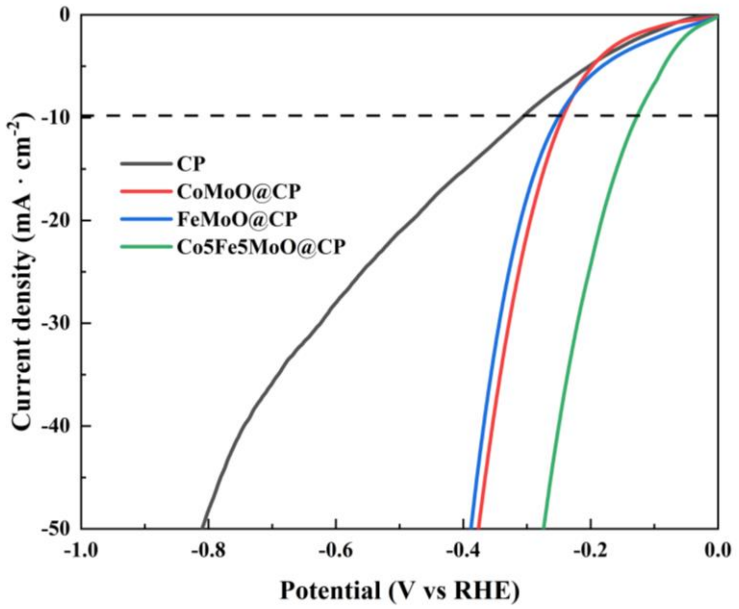

Fig. S23 The HER IR-corrected LSV polarization curves (detail) of CoMoO@CP, FeMoO@CP, and Co5Fe5MoO@CP materials

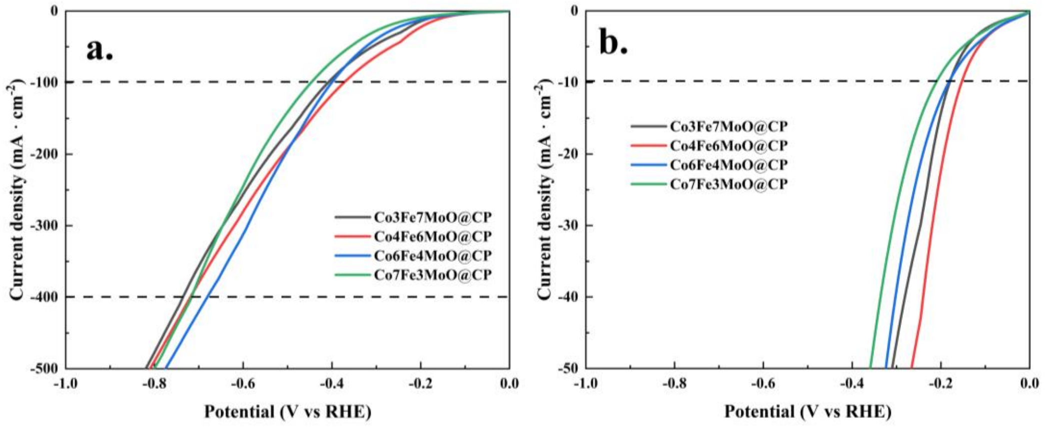

Fig. S24 The HER IR-corrected LSV polarization curves (a and b: detail) of CoxFe10-xMoO@CP materials

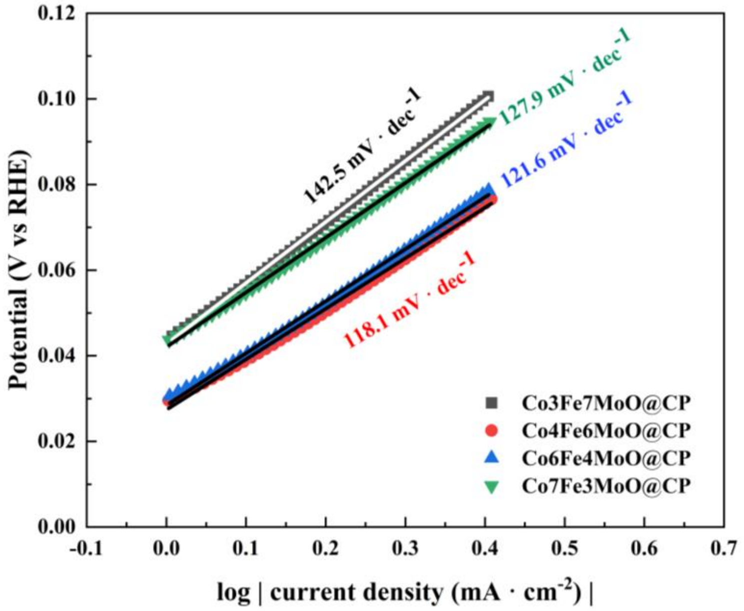

Fig. S25 The HER Tafel plots of CoxFe10-xMoO@CP materials

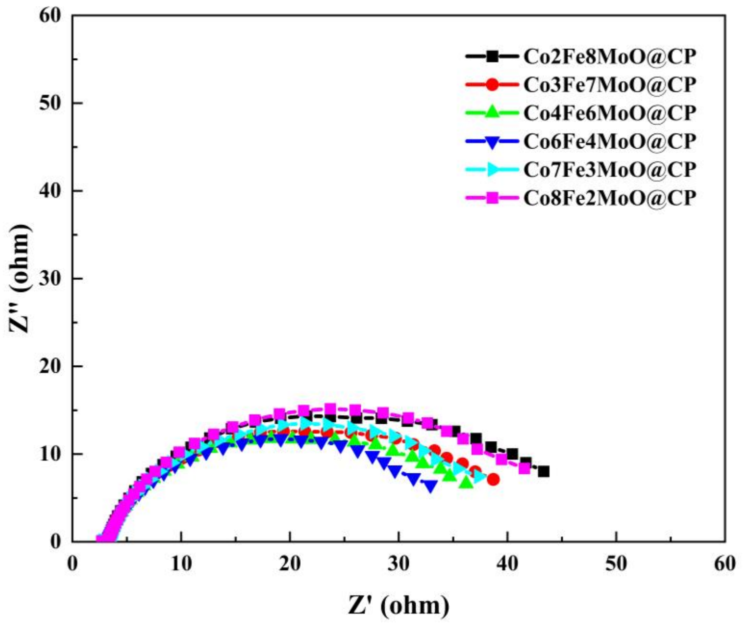

Fig. S26 The HER EIS spectra of CoxFe10-xMoO@CP materials

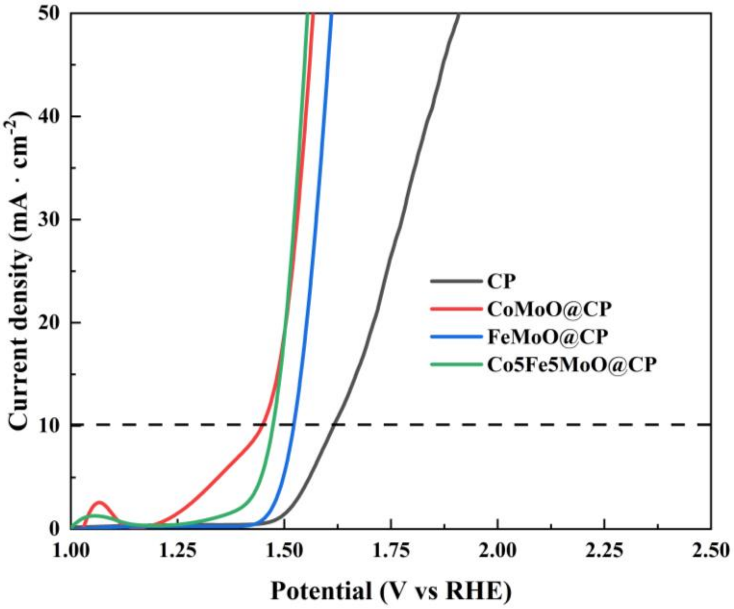

Fig. S27 The OER IR-corrected LSV polarization curves (detail) of CoMoO@CP, FeMoO@CP, and Co5Fe5MoO@CP materials

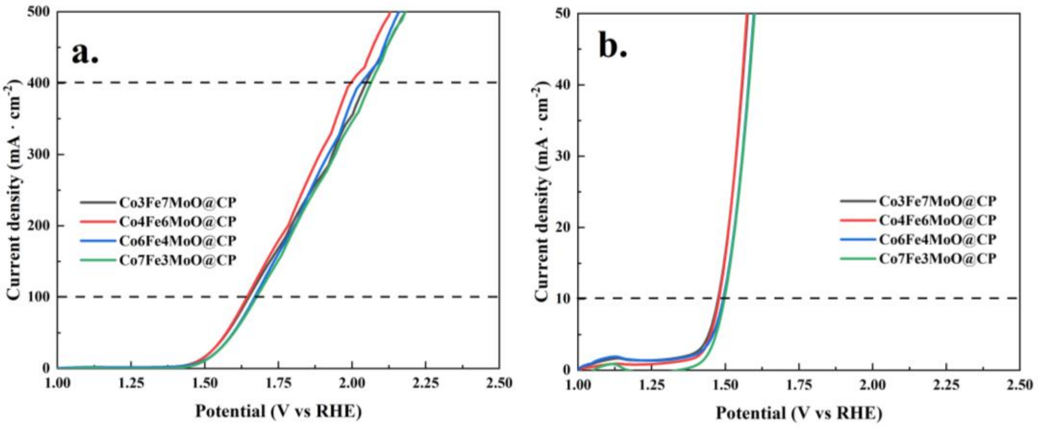

Fig. S28 The OER IR-corrected LSV polarization curves (a and b: detail) of CoxFe10-xMoO@CP materials

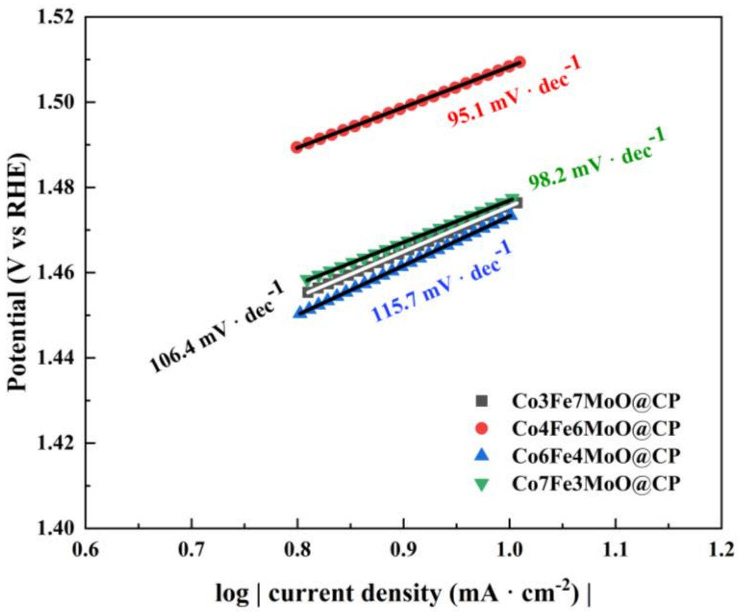

Fig. S29 The OER Tafel plots of CoxFe10-xMoO@CP materials

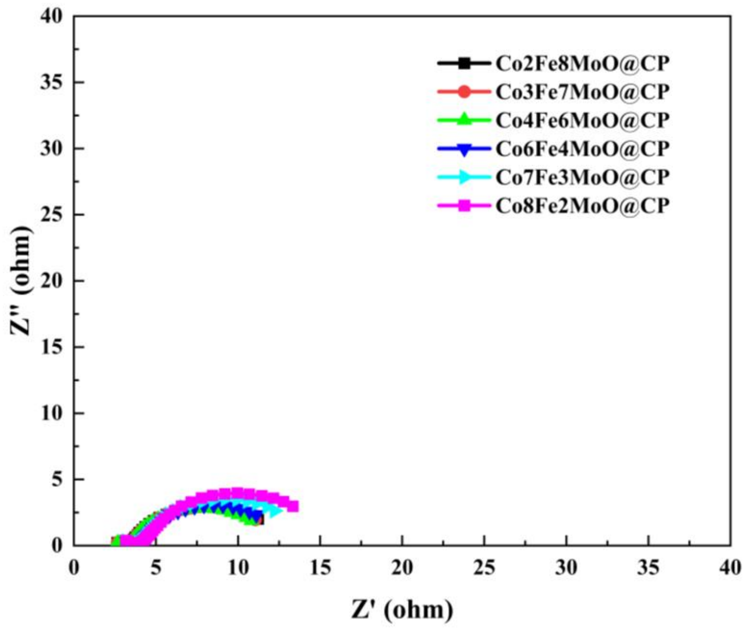

Fig. S30 The OER EIS spectra of CoxFe10-xMoO@CP materials

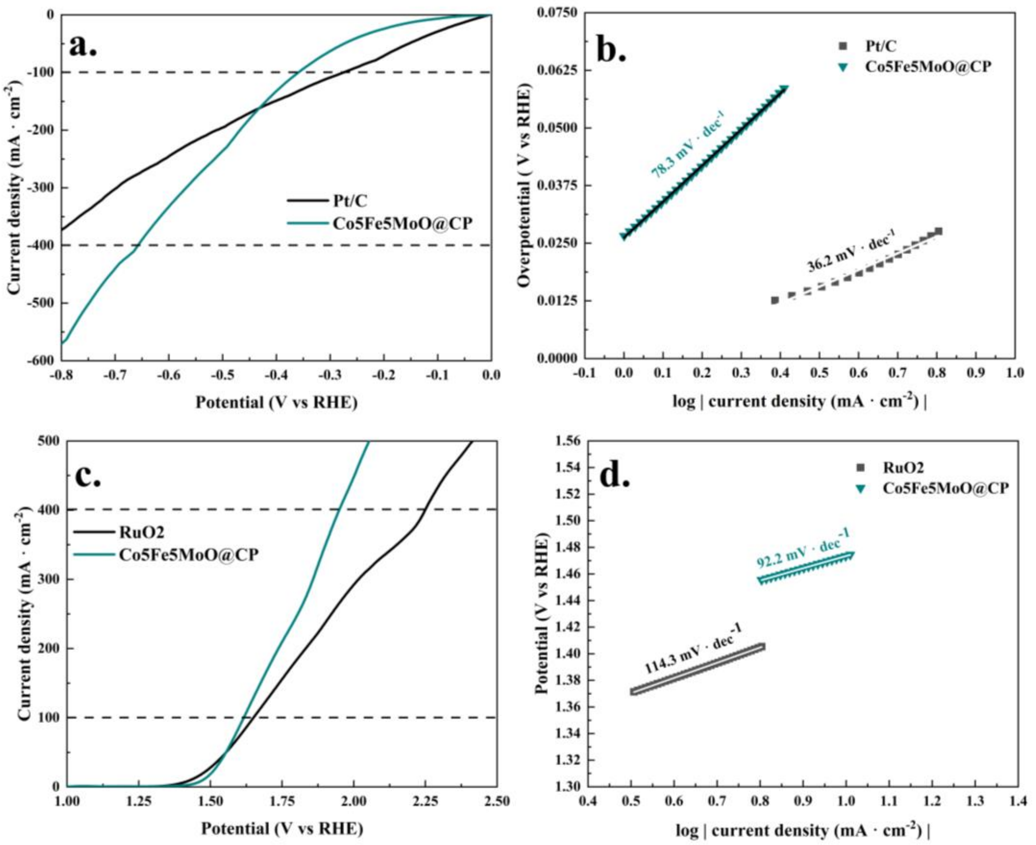

Fig. S31 The comparison of hydrogen and oxygen evolution efficiency between Co5Fe5MoO@CP material and commercial Pt/C and RuO_2 electrocatalysts (a and b: HER LSV and Tafel slope; c and d: OER LSV and Tafel slope)

S3. Table

Table S1 The overall water splitting performance of recent electrocatalytic materials

Catalyst	HER Overpotential (mV) at 10 mA·cm $^{-2}$	HER Tafel Slope (mV·dec^{-1})	OER Overpotential (mV) at 10 mA·cm $^{-2}$	OER Tafel Slope (mV·dec^{-1})	Medium	Reference
**MIL-101(Fe)/UiO-66**	185	178	290	144	1.0M KOH	[6]
**NiCoSe/C**	143	101	249	54	1.0M KOH	[7]
**Ni/MoC/$Ti_3C_2T_x$@C**	128	88	338	84	1.0M KOH	[8]
**FeCoNiP@NC**	187	51.7	266	35.6	1.0M KOH	[9]
**Mo_2NiB_2**	160	71	280	57	1.0M KOH	[10]
**NIM-175**	248	198	250	218	1.0M KOH	[11]
**FeCoNi-S_2**	80	45	280	49	1.0M KOH	[12]
**$CuCo_2S_4$@CoS_2**	153	151.7	261	89.2	1.0M KOH	[13]
**NiFeP@N-CS**	186	112	216	84	1.0M KOH	[14]
**SSFSe**	237	90	263	55	1.0M KOH	[15]
**Co5Fe5MoO@CP**	128	78.3	240	92.2	1.0M KOH	This work

References

[1] KRESSE G, FURTHMüLLER J. Efficient iterative schemes for ab initio total-energy calculations using a plane-wave basis set [J]. Physical Review B, 1996, 54(16): 11169-11186 doi: https://doi.org/10.1103/PhysRevB.54.11169.

[2] RAJAGOPAL A K, CALLAWAY J. Inhomogeneous Electron Gas [J]. Physical Review B, 1973, B7: 1912 doi: https://doi.org/doi.org/10.1103/PhysRevB.7.1912.

[3] KOHN W, SHAM L. Self-Consistent Equations Including Exchange and Correlation Effects [J]. Physical Review B, 1965, 140(4A): A1133 doi: https://doi.org/10.110310.1103/physrev.140.a1133.

[4] PERDEW J P, BURKE K, ERNZERHOF M. Generalized Gradient Approximation Made Simple [J]. Physical Review Letters, 1996, 77(18): 3865-3868 doi: https://doi.org/10.1103/PhysRevLett.77.3865.

[5] PERDEW J P, CHEVARY J A, VOSKO S H, JACKSON K A, PEDERSON M R, SINGH D J, FIOLHAIS C. Atoms, molecules, solids, and surfaces: Applications of the generalized gradient approximation for exchange and correlation [J]. American Physical Society, 1992, 48(11): 4978-4978 doi: https://doi.org/10.1103/PHYSREVB.46.6671.

[6] ALI M, PERVAIZ E. Fabrication of MIL-101(Fe) on UiO-66 as an Electrocatalyst for Enhanced Overall Water Splitting [J]. Chemistryselect, 2023, 8(37): e202301980 doi: https://doi.org/10.1002/slct.202301980.

[7] CHEN Z, XU B, YANG X, ZHANG H, LI C. Bimetallic metal-organic framework derived electrocatalyst for efficient overall water splitting [J]. International Journal of Hydrogen Energy, 2019, 44(12): 5983-5989 doi: https://doi.org/10.1016/j.ijhydene.2019.01.082.

[8] XU C, YANG X, FENG K, ZHANG M, YANG L, YIN S. Carbon-Encapsulated Multimetallic Hybrid Electrocatalyst for Overall Water Splitting and Urea Oxidation [J]. Acs Applied Energy Materials, 2023, 6(3): 1404-1412 doi: https://doi.org/10.1021/acsaem.2c03283.

[9] SUN J, LI S, ZHANG Q, GUAN J. Iron-cobalt-nickel trimetal phosphides as high-performance electrocatalysts for overall water splitting [J]. Sustainable Energy & Fuels, 2020, 4(9): 4531-4537 doi: https://doi.org/10.1039/d0se00694g.

[10] SAAD A, GAO Y, OWUSU K A, LIU W, WU Y, RAMIERE A, GUO H, TSIAKARAS P, CAI X. Ternary Mo2NiB2 as a Superior Bifunctional Electrocatalyst for Overall Water Splitting [J]. Small, 2022, 18(6): 2104303 doi: https://doi.org/10.1002/smll.202104303.

[11] NAGAJYOTHI P C, PAVANI K, RAMARAGHAVULU R, SHIM J. Microwave synthesis of NiMn2O4/Ni-foam: Efficient bifunctional electrocatalysts for overall water splitting [J]. International Journal of Hydrogen Energy, 2024, 54: 691-699 doi: https://doi.org/10.1016/j.ijhydene.2023.09.046.

[12] HE R, WANG C, FENG L. Amorphous FeCoNi-S as efficient bifunctional electrocatalysts for overall water splitting reaction [J]. Chinese Chemical Letters, 2023, 34(2): 107241 doi: https://doi.org/10.1016/j.cclet.2022.02.046.

[13] QIAN L-H, DONG W-W, CAO Y-B, MA R, DING Y, WANG X. ZIF-67-derived CuCo2S4@CoS2 as an efficient bifunctional electrocatalyst for overall water splitting [J]. New Journal of Chemistry, 2023, 47(44): 20574-20582 doi: https://doi.org/10.1039/d3nj03759b.

[14] HEI J, XU G, WEI B, ZHANG L, DING H, LIU D. NiFeP nanosheets on N-doped carbon sponge as a hierarchically structured bifunctional electrocatalyst for efficient overall water splitting [J]. Applied Surface Science, 2021, 549: 149297 doi: https://doi.org/10.1016/j.apsusc.2021.149297.

[15] TIAN Z, YANG L, WANG Z, XU C, LI D. Cost-effective polymetallic selenides derived from stainless steel foam (SSF) for overall water splitting [J]. Research on Chemical Intermediates, 2021, 47(11): 4779-4787 doi: https://doi.org/10.1007/s11164-021-04503-w.
